# Function of the *Pseudomonas aeruginosa* NrdR Transcription Factor: Global Transcriptomic Analysis and Its Role on Ribonucleotide Reductase Gene Expression

**DOI:** 10.1371/journal.pone.0123571

**Published:** 2015-04-24

**Authors:** Anna Crespo, Lucas Pedraz, Eduard Torrents

**Affiliations:** Bacterial Infections and Antimicrobial Therapies Group, Institute for Bioengineering of Catalonia (IBEC), Baldiri Reixac 15–21, 08028, Barcelona, Spain; University of Cape Town, SOUTH AFRICA

## Abstract

Ribonucleotide reductases (RNRs) are a family of sophisticated enzymes responsible for the synthesis of the deoxyribonucleotides (dNTPs), the building blocks for DNA synthesis and repair. Although any living cell must contain one RNR activity to continue living, bacteria have the capacity to encode different RNR classes in the same genome, allowing them to adapt to different environments and growing conditions. *Pseudomonas aeruginosa* is well known for its adaptability and surprisingly encodes all three known RNR classes (Ia, II and III). There must be a complex transcriptional regulation network behind this RNR activity, dictating which RNR class will be expressed according to specific growing conditions. In this work, we aim to uncover the role of the transcriptional regulator NrdR in *P*. *aeruginosa*. We demonstrate that NrdR regulates all three RNR classes, being involved in differential control depending on whether the growth conditions are aerobic or anaerobic. Moreover, we also identify for the first time that NrdR is not only involved in controlling RNR expression but also regulates topoisomerase I (*topA*) transcription. Finally, to obtain the entire picture of NrdR regulon, we performed a global transcriptomic analysis comparing the transcription profile of wild-type and *nrdR* mutant strains. The results provide many new data about the regulatory network that controls *P*. *aeruginosa* RNR transcription, bringing us a step closer to the understanding of this complex system.

## Introduction

The opportunistic pathogen *Pseudomonas aeruginosa* has the ability to grow under a variety of environmental conditions; it can be free-living in soil and water, as well as growing in human and plant host-associated environments. It is responsible for severe nosocomial infections in immunocompromised patients and, in particular, causes chronic lung infections in patients suffering from the inherited disease cystic fibrosis [1]. The genome of *P*. *aeruginosa* is relatively large (6.3 Mb), and contains a large number of genes to perform different metabolic activities, which might contribute to the environmental adaptability of this bacterium [2].

One such example is the enzyme ribonucleotide reductase (RNR), a key enzyme that catalyzes the reduction of all four ribonucleotides to their corresponding deoxyribonucleotides, providing the necessary precursors for DNA synthesis and repair in all organisms. All known RNRs can be divided into three classes (I, II and III) based on their structural differences, metallocofactor requirements, and mechanisms used for radical generation [3–6]. Class I RNRs require oxygen to produce a tyrosyl radical using a diferric iron or dimanganese iron center, and thereby functions only under aerobic conditions. Based on sequence identity, the metal cofactor center and allosteric properties, class I RNRs are subdivided into classes Ia, Ib and Ic, encoded, respectively, by *nrdAB*, *nrdHIEF* and the *nrdAB* genes. Class II enzymes require S-adenosylcobalamine (AdoCob) for radical generation and do not depend on oxygen. Members of class III RNR carry a stable but oxygen-sensitive glycyl residue plus an iron-sulfur center that catalyzes the reduction of S-adenosylmethionine to generate this radical. This class can only function under anaerobic conditions.

*P*. *aeruginosa* is one of the few microorganisms that encodes the three-different RNR classes (Class Ia, II and III) in its genome, which are apparently redundant, but reflect its need to adapt its metabolism to grow under specific conditions or during infection [7,8].

Relatively little is known about how bacteria control RNR activity at the gene level, and particularly in *P*. *aeruginosa*, in which it is totally unknown which transcriptional factors regulate the expression of the three RNR classes. The original study conducted by an Israeli group identified a novel transcriptional regulator in *Streptomyces coelicolor* termed NrdR, which controlled the expression of both class I and II RNR gene clusters. It was shown for the first time that in streptomycetes *nrdR* gene is linked to and co-transcribed with *nrdJ*. In *S*. *coelicolor*, a deletion of this gene produces a transcriptional derepression of the *nrd* genes [9,10]. Later, Rodionov and Gelfand described a bacterial regulatory system through a bioinformatics approach, with the identification of a highly conserved 16 bp palindromic signal, named NrdR-box, upstream of most operons encoding the ribonucleotide reductases [11]. Subsequently, our group described an analogous situation in *Escherichia coli*, with an NrdR homolog that was shown to regulate all three *nrd* systems (class Ia, Ib and III) and binding to the predicted NrdR binding sites. Remarkably, class Ib was highly derepressed (more than 150 times) in the *nrdR* mutant compared with the wild-type strain [12].

NrdR proteins are composed of 140–200 amino-acids, and present two differentiated domains: a zinc ribbon DNA-binding domain and an ATP-cone domain similar to that present in the N-terminal part (the allosteric activity site) of many RNRs. It seems that when the NrdR ATP-domain binds dATP instead of ATP, it changes its conformation and binds to its cognate DNA recognition sequences to repress RNR gene expression [10,13]. A recent study has unveiled a more complex control behind the NrdR nucleotide binding activity [14].

In this study, we uncovered the role of NrdR on the transcriptional regulation of the different ribonucleotide reductase and *topA* genes in *P*. *aeruginosa*. This is the first report in which the role of NrdR was analyzed in *P*. *aeruginosa* whose genome encodes all three different RNR classes. We also studied the global expression profile of *P*. *aeruginosa* when the *nrdR* gene was mutated, and the role of this transcription factor as a global regulator.

## Materials and Methods

### Bacterial strains, plasmids and growth conditions

Bacterial strains and plasmids are listed in Table 1. *Escherichia coli* and *Pseudomonas aeruginosa* cells were routinely grown in Luria-Bertani broth (LB) at 37ºC. When necessary, antibiotics were added at the following concentrations: for *E*. *coli*, 10 μg/ml gentamicin and 50 μg/ml ampicillin; for *P*. *aeruginosa*, 150 μg/ml gentamicin, 300 μg/ml carbenicillin and 50 μg/ml tetracycline. Liquid cultures were shaken on a horizontal shaker at 200 rpm. Anaerobic growth was performed in LB medium containing 10 g/l KNO_3_ in screw-cap tubes (Hungate Tubes) that were filled to the top with N_2_.

**Table 1:** Bacterial strains and plasmids used in this study.

Strain or plasmids	Description	Source
**Plasmids**		
pGEM-T easy	A/T cloning vector, Amp^R^	Promega
pUCP20T	Broad-host-range vector, Amp^R^	[32]
pBBR1MCS-5	High-copy number cloning vector, Gm^R^	[33]
pETS130-GFP	Broad host range, promoterless GFP, Gm^R^	[7]
pETS134	pETS130 derivative carrying *nrdA* promoter, Gm^R^	[7]
pETS136	pETS130 derivative carrying *nrdD* promoter, Gm^R^	[7]
pETS161	pETS130 derivative carrying *nrdR* promoter, Gm^R^	This work
pETS176	pUCP20T derivative carrying *nrdR* gene, Amp^R^	This work
pETS177	pETS130 derivative carrying *topA* promoter, Gm^R^	This work
pETS178	pETS130 derivative carrying NrdR box mutated in *topA* promoter, Gm^R^	This work
pETS180	pETS130 derivative carrying *nrdJ* promoter, Gm^R^	This work
pETS181	pETS130 derivative carrying NarL box1.1 mutation (box NarL1) in *nrdR* promoter, Gm^R^	This work
pETS182	pETS130 derivative carrying NarL box1.2 mutation (box NarL1) in *nrdR* promoter, Gm^R^	This work
pETS183	pETS130 derivative carrying NarL box1.3 mutation (box NarL1) in *nrdR* promoter, Gm^R^	This work
pETS184	pETS130 derivative carrying NarL box2.1 mutation (box NarL2) in *nrdR* promoter, Gm^R^	This work
pETS185	pETS130 derivative carrying NarL box2.2 mutation (box NarL2) in *nrdR* promoter, Gm^R^	This work
pETS186	pETS130 derivative carrying NarL box2.3 mutation (box NarL2) in *nrdR* promoter, Gm^R^	This work
pETS187	pETS130 derivative carrying NarL box1 and box2 mutation in *nrdR* promoter, Gm^R^	This work
pETS188	pETS130 derivative carrying NrdR box2 mutated in *nrdA* promoter, Gm^R^	This work
pETS189	pETS130 derivative carrying NrdR box2 mutated in *nrdJ* promoter, Gm^R^	This work
pETS190	pETS130 derivative carrying NrdR box2 mutated in *nrdD* promoter, Gm^R^	This work
**Strains**		
*E*. *coli*		
DH5α	*recA1 endA1 hsdR17 supE44 thi-1 relA1 Δ(lacZYA-argF)U169 deoR Φ80dlacZM15*	Laboratory stock
*P*. *aeruginosa*		
PAO1	Wild-type (ATCC 15692 / CECT 4122)- Spanish Type Culture Collection	Lab strain
PW7549	*P*. *aeruginosa* PAO1 *narL*::*IS*l*acZ*/hah, Tc^R^	[20]
PW7855	*P*. *aeruginosa* PAO1 *nrdR*::*IS*l*acZ*/hah, Tc^R^	[20]

### Strains and plasmids construction

Recombinant DNA manipulations were carried out according to published protocols [15]. Plasmid DNA was prepared using the QIAprep miniprep kit (Qiagen) and was transformed into *P*. *aeruginosa* cells by electroporation as previously described [16] using a Gene Pulser Xcell^TM^ electroporator (Bio-Rad). Digestions with restriction enzymes were performed according to the manufacturer’s instructions (Fermentas). Ligations were performed with T4 DNA ligase (Fermentas, Thermo Scientific), except as otherwise stated. DNA fragments were amplified by PCR using High-Fidelity PCR enzyme mix (Fermentas) using chromosomal DNA as a template.

When necessary, specific restriction site sequences were incorporated at the 5’ ends of the primers to facilitate the cloning of the fragments in the appropriate vector. Plasmids pETS161, pETS177 and pETS180 were constructed as follows: First, the *nrdR* (277 bp), *topA* (348 bp) and *nrdJ* (419 bp) promoter regions were amplified from *P*. *aeruginosa* PAO1 genomic DNA using the primer pair P*nrdRBHI*-up/P*nrdRClaI*-lw; PtopA-BamHI-up/PtopA-ClaI-low; and PnrdJ-BamHI new-up/PnrdJSmaI-new-low, respectively (S1 Table). The resulting DNA fragment and the pETS130-GFP plasmid were both digested with the corresponding restriction enzymes, and ligation was performed. Complementation plasmid (pETS176) was constructed by cloning the *nrdR* gene, under the control of its native promoter, into plasmid pUCP20T using the primer pair P*nrdRBamHI*-up/NrdRHindIII-low.

### Site-directed mutagenesis of the putative NarL and NrdR binding sites

The two NarL binding boxes (NarL1 and NarL2) in the *nrdR* promoter region were mutated using PCR-based site-directed mutagenesis using the following primer pairs: mutNarL1up/mutNarL1low; mutNarL1.2 up/mutNarL1.2 low; mutNarL1.3 up/mutNarL1.3 low; mutNarL R-dir/mutNarL-rev; mutNarL2.2 up/mutNarL2.2 low; and mutNarL2.3 up/mutNarL2.3 low, to generate pETS181, pETS182, pETS183, pETS184, pETS185, pETS186 and pETS187, respectively.

The putative NrdR box2 in the promoter regions of *nrdAB*, *nrdJ*, *nrdDG* and *topA* was mutagenized using the following primer pairs: AmR2-up/AmR2-low; JmR2-up/JmR2-low; DmR2-up/DmR2-low; and TmR-up/TmpR-low, to generate pETS188, pETS189, pETS190 and pETS178, respectively. The resulting amplicons were cloned into the pGEM-T easy vector, according to the manufacturer’s instructions, and then, after digestion with the corresponding restriction enzymes, to pETS130-GFP. Each mutation was verified by DNA sequencing.

### Green fluorescent protein gene reporter assay

Bacterial cultures were grown to the corresponding A_550_, and three independent 1-ml samples of each culture were collected. Cells were pelleted, and fixed with 1 ml of freshly prepared phosphate buffered saline (PBS) solution containing 2% formaldehyde and stored in the dark at 4ºC. Fluorescence was measured in 96-well plates on an Infinite 200 Pro fluorescence microplate reader (Tecan). Three measurements were performed for each independent sample.

### DPA assay

For total cellular dNTP quantification we used the diphenylamine assay (DPA) following the described procedures [17,18]. Briefly, DPA reagent (Sigma-Aldrich) was dissolved in a 2:1 acetic acid–sulfuric acid mixture. The solution was incubated at 37ºC for 4 h, and all measurements were performed at 595 nm. Bacterial cell extracts from *P*. *aeruginosa* wild-type cells grown to an A_550_ of 0.5 and normalized by equal protein content were analyzed using the DPA assay. Three independent experiments were performed.

### Supercoiling assay

pUCP20T plasmid was transformed into PAO1 wild-type and *nrdR* mutant strains by electroporation, to corroborate differences in supercoiling activity. Strains were grown aerobically at 37ºC to mid-logarithmic and stationary phases (A_600_ of 0.5 and 2, respectively) in LB containing 300 μg/ml of carbenicillin. Plasmid DNA was purified via a previously described protocol [19]. Briefly, a 16 h gel electrophoresis at 50 V was performed in 1.2% agarose gels containing 5 mg/L of chloroquine, to separate 0.5 μg of plasmid. After washing for 3 h in water, to remove chloroquine, the gels were stained with ethidium bromide and visualized on an ultraviolet transilluminator.

### RNA extraction, reverse transcription and real-time PCR

Total RNA from *P*. *aeruginosa* PAO1 was isolated with an RNeasy Mini Kit (Qiagen) and RNAprotect Bacteria Reagent (Qiagen), according the manufacturer’s instructions. DNase I (Turbo DNA-free, Applied Biosystems) was used to remove DNA contamination. Reverse transcription PCR (RT-PCR) was performed with 1 μg of RNA in a total 20-μl reaction volume, using the SuperScript III First-Strand Synthesis System for RT-PCR (Applied Biosystems), and PCR amplification of the cDNA was performed with High-Fidelity PCR enzyme mix (Fermentas). Primers used in this study are listed in S1 Table. The first-strand cDNA synthesis step was conducted at 55ºC for 1 h, and the cycling conditions for PCR were performed as follows: 3-min denaturation period at 94ºC; 20 cycles for 1 min at 94ºC, 45 s at 51ºC, and 1 min per kb of DNA template at 72ºC; and final 7-min extension at 72ºC.

Real-Time PCR measurements were carried out using TaqMan primers and probes (S1 Table), and detection was performed using and ABI Step One Plus detection system from Applied Biosystems as described previously [12]. The *gapA* sequence was used as an internal standard since their expression is constitutive during *P*. *aeruginosa* growth.

### Microarray analysis

The *P*. *aeruginosa* strains were grown aerobically and anaerobically until the mid-logarithmic growth phase. Total bacterial RNA was isolated as previously stated from each of three independent cultures. Eight micrograms of purified RNA were used for a GeneChip genome array analysis. The GeneChip probes (Affymetrix) were prepared according to Affymetrix’s instructions. RNA integrity, target hybridization, washing, staining and scanning steps were performed by the Functional Genomics Core facility at the Institute for Research in Biomedicine (Barcelona, Spain). Data analysis was initially performed with the Microarray suite software and then imported into Microsoft Excel for further statistical analysis. Only those genes that had a mean signal log_2_-ratio of >1.5 (up-regulated transcripts) and <1.5 (down-regulated transcripts) were kept in the final list of genes. Microarray data are available in the Array express database (www.ebi.ac.uk/arrayexpress) under accession number E-MTAB-3006.

### Fly infection assays

All experiments used healthy 3–4 days old adult *Drosophila melanogaster* Oregon^R^ flies, maintained at 25ºC in vials with standard corn-meal agar medium. A suspension of *P*. *aeruginosa* cells in PBS, adjusted at A_550_≈0.1, was injected using a capillary glass with a microinjector (TriTEch Research, CA) as previously described [7]. Survival curves were plotted using Kaplan-Meier analysis and differences of survival rates were analyzed by the log-rank test (GraphPad Prism 6.0, GraphPad Software, La Jolla California USA).

## Results

### NrdR expression pattern in *P*. *aeruginosa*

**Fig 1:**
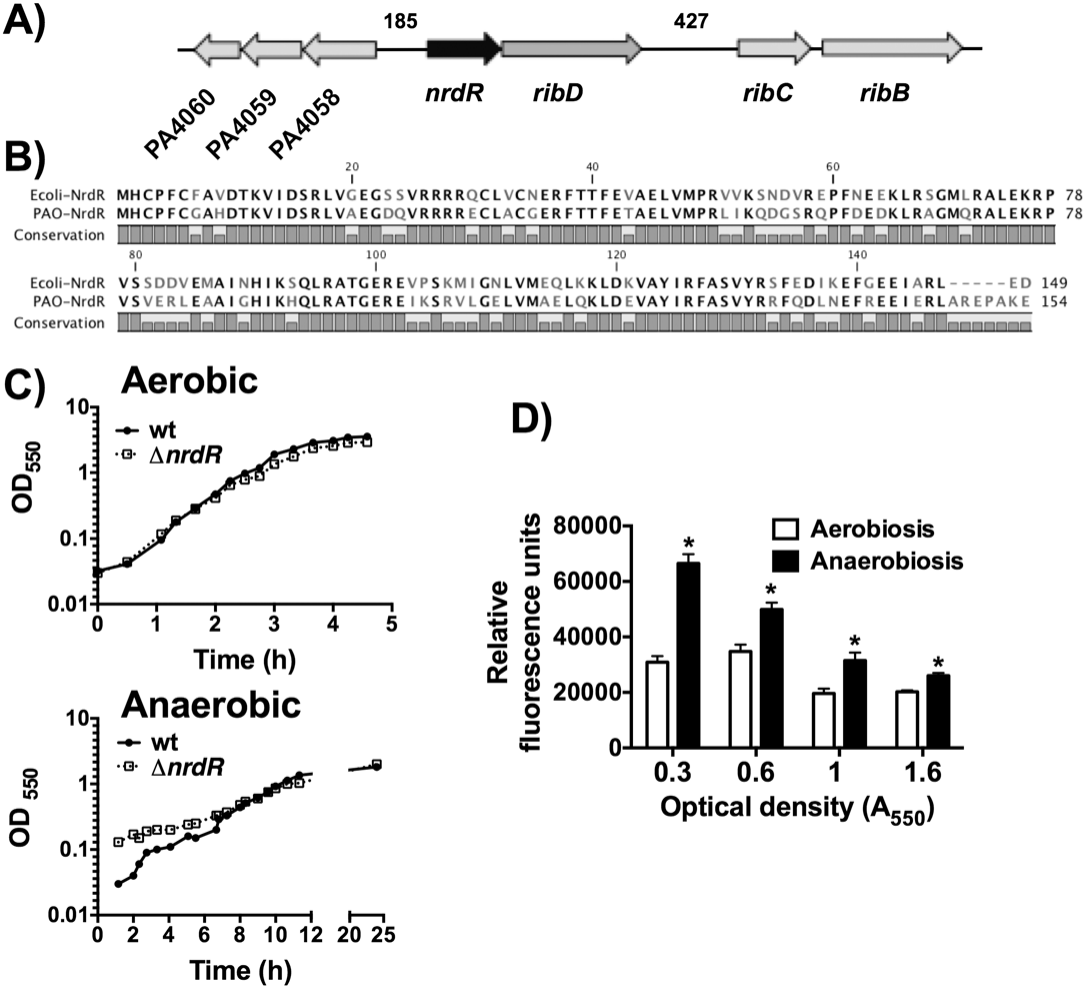
*nrdR* operon organization and expression. **A)** Gene organization scheme of the *nrdR-rib*D operon. **B)** Sequence alignment (Clustal W) of *P*. *aeruginosa* (PAO-NrdR; Uniprot Q9HWX1) and *Escherichia coli* (Ecoli-NrdR; Uniprot P0A8D0) NrdR proteins. **C)** Aerobic and anaerobic growth curve of *P*. *aeruginosa* strains PAO1 (wild-type) and PW7855 (Δ*nrdR*). **D)** Fluorescence (GFP) was measured in both strains harboring pETS161 (P*nrdR*-GFP) at different points of growth, at 37ºC in LB medium. The fluorescence was normalized dividing by the optical density (A_550_), and it is given in relative fluorescence units. Each experiment was repeated three times, and the results are the mean ± standard deviation. *: Significantly different compared with wild-type strain in an unpaired *t*-test (*P*<0.05).

The *P*. *aeruginosa* transcriptional regulator homolog of the *E*. *coli nrdR* gene is the PA4057 gene (Fig 1A). The translation of the PA4057 gene, here denoted as *nrdR*, is expected to produce a 154 amino-acid protein, with a predicted molecular weight of 17.9 kDa. A search in the Conserved Domain Database revealed two major domains: a zinc-finger (3–34 aa) and an ATP-cone domain (49–139 aa), at the N-terminus and C-terminus, respectively, showing a structure similar to all NrdR proteins [13]. In a 149 amino-acid overlap, *P*. *aeruginosa* NrdR showed 70% identity and 82% similarity with *E*. *coli* NrdR (see alignment in Fig 1B).

Contrary to the observation in the initial IS insertion *nrdR* mutant in *E*. *coli* [12], a *P*. *aeruginosa nrdR* mutant showed a similar growth curve to that of the wild-type strain, under both aerobic and anaerobic conditions (Fig 1C). The insertion sequence element disrupting the *nrdR* gene (strain PW7855, from now on Δ*nrdR*) contains an internal promoter that allows the expression of downstream genes, as indicated by the authors [20].

As shown in Fig 1D, the transcriptional fusion of the *nrdR* promoter to the green fluorescent protein (GFP) (see materials and methods) revealed an increased *nrdR* expression in exponential phase and a decrease in stationary phase, under both aerobic and anaerobic growing conditions. Clearly, the NrdR protein is expressed at higher levels during the exponential growth phase, particularly under anaerobic conditions.

### NarL is responsible for the anaerobic expression of the *nrdR* gene

**Fig 2:**
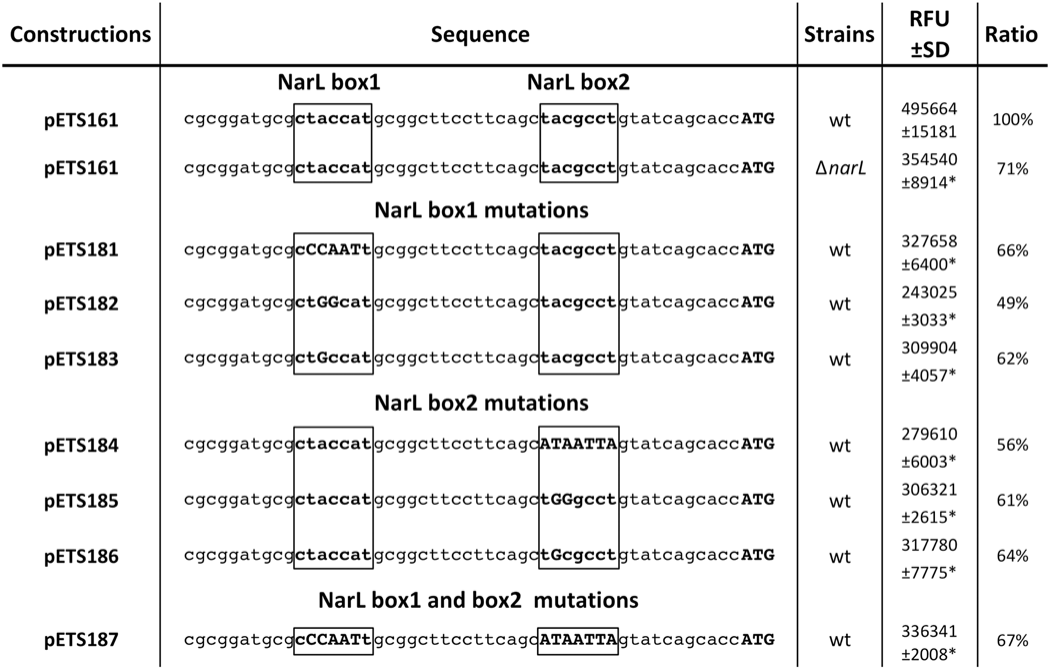
NarL-dependent expression of *nrdR*. **A)** Representation of the *P*. *aeruginosa* PAO1 *nrdR* promoter region sequence, indicating the different mutated NarL binding sites. Black boxes indicate the putative NarL recognition sites, and mutated sequences are shown in upper case and in bold letters. The transcription start site is indicated in bold. The RFU column shows the relative fluorescence intensity presented by the *P*. *aeruginosa* wild-type *nrdR* promoter fusion (pETS161), compared with their mutated NarL boxes (pETS181, pETS182 and pETS183 for NarL box1, pETS184, pETS185 and pETS186 for NarL box2, and pEST187 harboring the double mutation). The expression of wild-type *nrdR* promoter under a Δ*narL* mutant background is also stated. The ratio column shows a comparison of all the conditions with the expression of a wild-type promoter under a wild-type background. Strains were grown anaerobically until the mid-logarithmic phase. Values represent the mean of three independent experiments. Transcriptional start codon is shown in bold. Three independent experiments were performed and the mean±standard deviation is shown). *: Significantly different compared with wild-type promoter region (pETS161) in an unpaired *t*-test (*P*<0.05).

We investigated the molecular mechanism that carries out the transcriptional activation of the *nrdR* gene under anaerobic conditions. An initial examination of the *nrdR* promoter region, using the Virtual Footprint tool from the PRODORIC database [21], revealed two heptameric NarL-binding sites located at 18 and 40 bp upstream of the translation start codon, denoted here as NarL1 (CTACCAT) and NarL2 (TACGCCT) boxes (Fig 2). To confirm the bioinformatical prediction, the two putative heptameric NarL-binding sites (NarL1 and NarL2 boxes) were mutated.

Three mutations were performed in each box, focusing on the most important nucleotides according to the published *P*. *aeruginosa* NarL consensus binding sequence (TAC^C^/_T_N^A^/_C_T) [22]. Therefore, plasmids harboring the different mutations in NarL1 and NarL2 boxes were made (pETS181 to pETS187, see Table 1). A decrease of the promoter expression under anaerobic conditions was observed, compared with the wild-type promoter (pETS161) (Fig 2, S1 Fig). The activities obtained when mutating NarL1 and NarL2 boxes were similar to those obtained for the wild-type promoter region (pETS161) in the *narL* knockout strain (PW7549; from now on Δ*narL*) (Fig 2). These results confirm a direct activation of the *nrdR* expression via binding of NarL.

### NrdR regulates the expression of the three different ribonucleotide reductase classes

To study whether the NrdR protein regulates the expression of the different *nrd* genes, we measured the expression of the different *nrd* promoters in *P*. *aeruginosa* wild-type and a Δ*nrdR* mutant strain (PW7855), using plasmids carrying a transcriptional fusion of each RNR promoter region and the *gfp* reporter gene (see materials and methods, and [7]).

**Fig 3:**
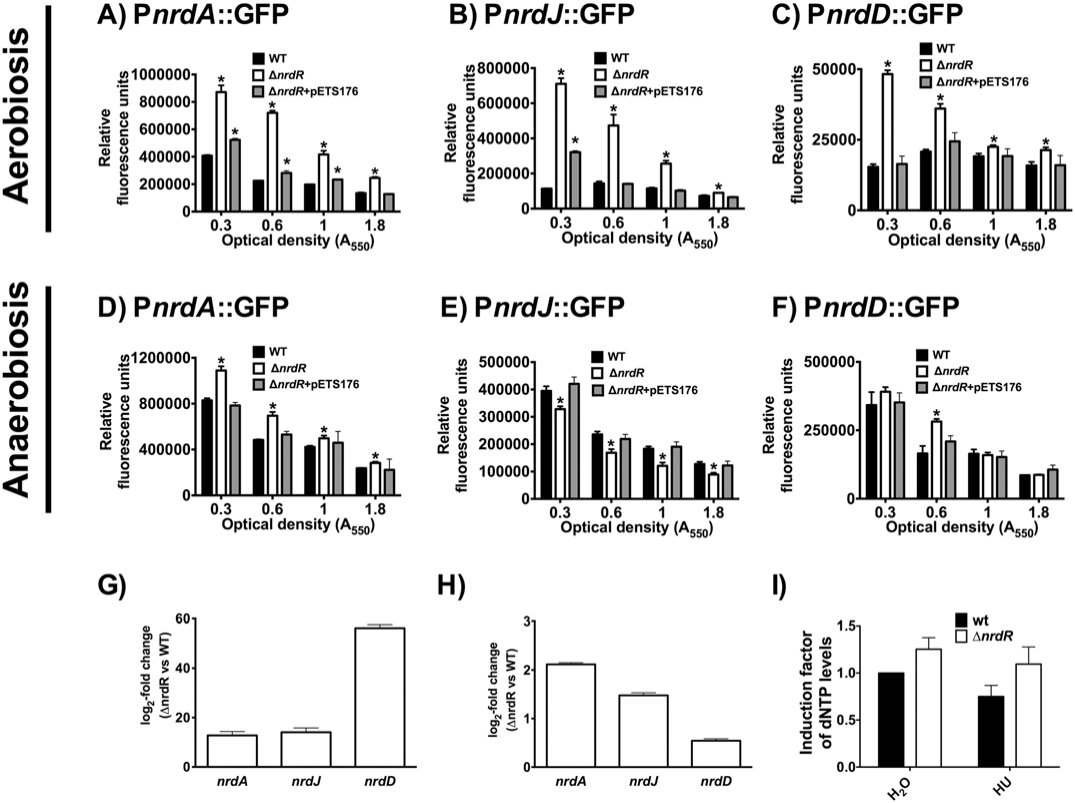
NrdR differentially regulates ribonucleotide reductase genes in aerobiosis or anaerobiosis. Aerobic expression studies are shown in **A-C** and **G**, and anaerobic expression studies in **D-F** and **H**. *P*. *aeruginosa* wild-type strain (black bars), Δ*nrdR* mutant strain (white bars) and the deficiency-complemented *nrdR* strain (Δ*nrdR+*pETS176) (gray bars) bearing the promoter fusions P*nrdA*-*gfp* (panel **A** and **D**), P*nrdJ*-*gfp* (panel **B** and **E**) and P*nrdD*-*gfp* (panel **C** and **F**), were grown as indicated in the material and methods. GFP fluorescence is expressed as arbitrary units subtracting the reads of the control plasmid pETS130. **G**) and **H**) Quantitative RT-PCR analysis of genes encoding three different classes of RNR. qRT-PCR was conducted on cDNA synthesized from wild-type, compared with Δ*nrdR* cells, both grown aerobically (A_550_ = 0.6) (**G**) and anaerobically (A_550_ = 0.6) (**H**). The means of three independent experiments are displayed, and the error bars represent the positive standard deviation **I)** dNTPs pool level of aerobic *P*. *aeruginosa* wild-type and *nrdR* mutant cells treated with 10 mM hydroxyurea (HU), measured by DPA assay. DNA contents were normalized with those of wild-type strain. Three independent experiments were performed and the mean ± standard deviation is shown. *, Significantly different compared with the wild-type strain in an unpaired *t*-test (*P*<0.05).

Under aerobic conditions (Fig 3A–3C), all three *nrd* genes (*nrdA*, *nrdJa* and *nrdD*) showed an evident increase in their expression (from 3 to 6-fold) in the *nrdR* mutant, compared with the wild-type strain, indicating that NrdR acts as its repressor. The maximal difference appeared in the transcription of the class II RNR (*nrdJa*). Note that the aerobic transcription of the *nrdJa* and *nrdD* genes was approximately 8–10 times lower compared with the *nrdA* gene and highly expressed under anaerobic conditions in the wild-type strain, compared to aerobic conditions.

A completely different expression pattern was observed under anaerobic conditions (Fig 3D–3F). Expression of the *nrdA* gene slightly increased in the *nrdR* mutant (1.3-fold, Fig 3D). Expression of *nrdJa* is down-regulated in the *nrdR* mutant, and no change in *nrdD* expression was observed compared with the wild-type strain (Fig 3E and 3F). Under all conditions, complementation with the *nrdR* gene cloned into plasmid pUCP20T (pETS176) returned the expression to the wild-type level.

One of the two putative NrdR boxes that were identified in all RNRs promoters (S3 Fig) was mutated by PCR-based site-directed mutagenesis. Plasmids harboring the mutant promoter confirmed our previous results, hence indicating the functionality of the NrdR boxes (S2 and S3 Figs).

To correlate the transcriptional data with mRNA quantity, we measured the levels of mRNA for each RNR class in wild-type cells and Δ*nrdR* mutant strain by real-time PCR (Fig 3G and 3H) at mid-logarithmic phase (A_550_ = 0.6). Aerobically, (Fig 3G) all RNR genes were highly expressed (from 13 to 56 times) in the *nrdR* mutant compared with the wild-type. By contrast, anaerobically (Fig 3H), the *nrdA* gene slightly increased its expression (2.1 times), and no effect was observed on the transcription of the *nrdJa* and *nrdD* genes in the *nrdR* mutant compared with the wild-type strain, corroborating our transcriptional fusion expression results.

As expected, when we inactivated the *nrdR* gene, the dNTP pool levels observed were 25% higher compared with the wild-type strain (Fig 3I), suggesting that we eliminated the NrdR repressor, and, therefore, increased global RNR activity under all conditions.

Finally, we aimed to address the effect of the NrdR regulation at a physiological level by changing the levels of dNTPs as seen by other authors [23,24]. Hydroxyurea is a known radical scavenger that inhibits class Ia RNR catalytic activity, thus reducing the amounts of dNTPs. When 10 mM hydroxyurea were added to the medium during aerobic growth, the dNTP amounts were 25% lower. This reduction could be restored in an *nrdR* mutant strain (enhancing class II RNR activity) which returns to wild-type dNTPs levels (Fig 3I).

### NrdR represses *topA* expression

All genes that have been described as transcriptionally regulated by NrdR were ribonucleotide reductase encoding genes. Rodionov and Gelfand [11] and recent databases (RegPrecise; http://regprecise.lbl.gov/RegPrecise/index.jsp) highlighted the possible implication of NrdR in the regulation of the *P*. *aeruginosa* DNA topoisomerase I gene *topA* (PA3011), identifying a single putative NrdR box in its promoter region (see S3 Fig).

**Fig 4:**
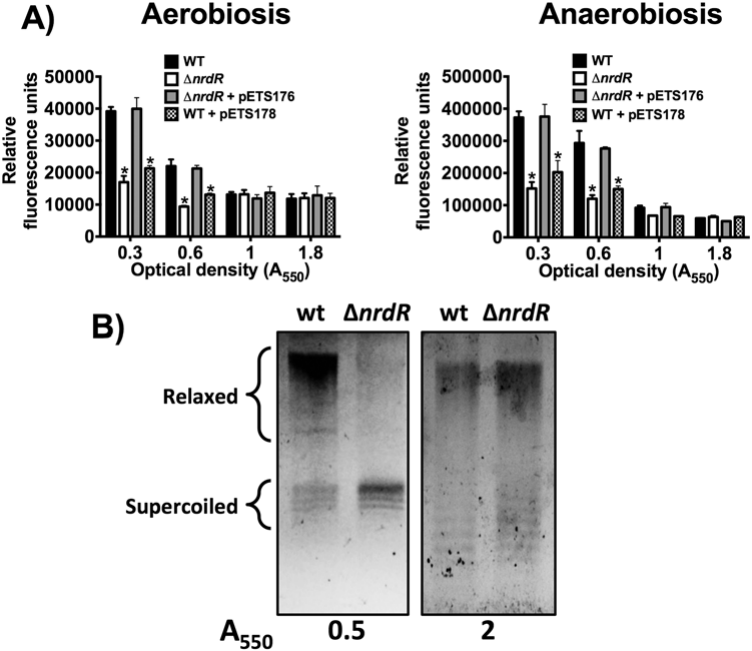
*topA* expression is activated aerobically and anaerobically by NrdR. **A)** GFP fluorescence was measured in *P*. *aeruginosa* strains PAO1 (wild-type) and PW7855 (Δ*nrdR*) harboring plasmid pETS177 (P*topA*::GFP). The *nrdR* cloned into plasmid pUCP20T (pETS176) was used to complement *nrdR* deficiency in strain PW7855. Plasmid pETS178 harbors a *topA* promoter with a mutation in the NrdR box. The fluorescence was normalized with the absorbance at 550 nm (A_550_) and it is given in relative fluorescent units. The bars represent the mean of three independent experiments ± standard deviation. **B)** A gel electrophoresis assay, in an agarose gel containing chloroquine, of plasmid DNA isolated from *P*. *aeruginosa* wild-type and Δ*nrdR* strains, at mid-logarithmic and stationary phases. The direction of migration was from top to bottom. *, Significantly different compared with the wild-type strain in an unpaired *t*-test (*P*<0.05).

Expression of the *topA* gene during exponential growth under aerobic or anaerobic conditions was repressed in the *nrdR* mutant (2–3 times) compared with the wild-type strain, suggesting that NrdR acts as a *topA* activator during the exponential growth phase (Fig 4).

Complementation with an extra *nrdR* gene (pETS176) returned the *topA* expression level to the wild-type levels. When the NrdR binding box was mutated in the promoter *topA* region (pETS178) the expression levels were similar to the levels found in the Δ*nrdR* strain, therefore corroborating the functionality of the unique NrdR-binding region on the *topA* promoter region (Fig 4, S3 Fig).

The degree of supercoiling in bacterial DNA is determined by the balance between DNA-relaxing activity and DNA-supercoiling activity, regulated by the opposing actions of topoisomerase I encoded by the *topA* gene and DNA gyrase, respectively [19]. The prokaryotic topoisomerase I is only capable of relaxing negatively supercoiled DNA. To phenotypically corroborate the *topA* down-regulation in the *P*. *aeruginosa* Δ*nrdR* strain, we analyzed the DNA topology of pUCP20T in an electrophoresis assay in an agarose gel with chloroquine. pUCP20T plasmid extracted from the Δ*nrdR* mutant strain showed more negative supercoiled DNA compared with the wild-type strain (Fig 4B) when measured during exponential growing phase. According to the gene reporter assay, no difference in supercoiled DNA levels appears during stationary growing phase. This result reflects a possible change in DNA topology in the *P*. *aeruginosa* Δ*nrdR* strain, compared with the wild-type strain.

### Global gene expression profiling of the *P*. *aeruginosa nrdR* mutant strain compared with the wild-type strain

We previously showed that NrdR directly regulates the three *P*. *aeruginosa* RNR classes and topoisomerase I (*topA*), all of which are involved in bacterial DNA replication. To determine the global transcriptional changes produced by a mutation in *nrdR*, we initiated a gene profiling experiment using the Affymetrix *P*. *aeruginosa* GeneChip microarray platform.

RNA was isolated from a *P*. *aeruginosa* PAO1 wild-type strain and a Δ*nrdR* mutant strain, both grown aerobically and anaerobically in LB medium to mid-logarithmic growth phase. Labeled RNA was hybridized to Affymetrix *P*. *aeruginosa* GeneChips and gene expression levels between Δ*nrdR* mutant and wild-type strains were compared.

**Fig 5:**
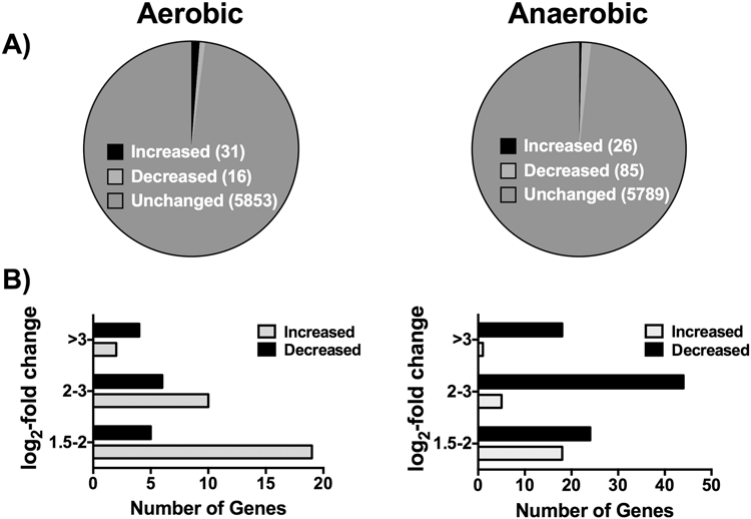
Summary of the effects of the *nrdR* mutation on *P*. *aeruginosa* gene expression under aerobic and anaerobic conditions. **A)** Distribution of the different genes (up-regulated, down-regulated and unchanged) in gene expression (>1.5 Log_2_ fold change). The number of gens in each category is indicated. **B)** Distribution of genes whose expression was either increased or decreased in a Δ*nrdR* mutant strain, grouped according to fold-changes in expression levels.

Results showed altered transcription levels in only a few genes, comparing the Δ*nrdR* mutant strain to the wild-type strain. Aerobically only 47 genes (0.8%) were significantly deregulated, with 31 genes up-regulated (0.5%) and 16 genes down-regulated (0.3%). Anaerobically, 111 genes were differentially regulated, with 26 genes up-regulated (0.45%) and 85 genes down-regulated (1.45%). Only few genes were expressed or repressed more than 3 log_2_ fold change (Fig 5B). To corroborate our array a selection of some deregulated genes was measured by quantitative PCR (S5 Fig).

Under aerobic conditions, among the most up-regulated genes we found the fimbrial subunit *cupA1* (log_2_-fold change of 4.13), several hypothetical proteins (PA4139, PA1383 and PA2223 with log_2_-fold changes of 3.78, 2.42 and 2.37, respectively) and antibiotic resistance related genes, such as the entire *mexAB-orpM* operon (log_2_-fold change from 2.13 to 1.43) (Table 2 and S2 Table). In addition the RNR genes were found to be up-regulated, as expected (log_2_-fold changes from 2.41 and 1.96). The highest repression under this condition was found in several hypothetical proteins (PA3283, PA3281, PA0565 with log_2_-fold changes of -4.57, -3.73, -3.06) and also several genes involved in antibiotic resistance, such as the entire *mexEF-oprN* operon (log_2_-fold change from -3.19 to -1.13).

**Table 2:** Global transcriptomic analysis of a Δ*nrdR* mutant strain compared with the *P*. *aeruginosa* PAO1 wild-type strain.

ID	*Gene*	*Operon arrangement*	Log2 Fold-Change	*Gene Product*
**Aerobic**				
PA2128	*cupA1*	*cupA12345*	4.13	Fimbrial subunit CupA1
PA1383			2.42	Hypothetical protein
PA4139			3.78	Hypothetical protein
PA5497	*nrdJa*	*nrdJab*	2.41	Class II (cobalamin-dependent) ribonucleotide-diphosphate reductase subunit, NrdJa
PA1718	*pscE*	*pscBCDEFGHIJKL*	2.32	Type III export protein PscE
PA1156	*nrdA*	*nrdAB*	2.24	Ribonucleoside reductase, large chain
PA0992	*cupC1*	*cupC123*	2.19	Fimbrial subunit CupC1
PA0425	*mexA*	*mexAB-oprM*	2.13	Resistance-Nodulation-Cell Division (RND) multidrug efflux membrane fusion protein
PA0424	*mexR*		2.06	Multidrug resistance operon repressor MexR
PA1693	*pscR*	*PA1697-pscOPQRSTU*	2.00	Translocation protein in type III secretion
PA1155	*nrdB*	*nrdAB*	1.96	Ribonucleoside reductase, small chain
PA0426	*mexB*	*mexAB-oprM*	1.93	Resistance-Nodulation-Cell Division (RND) multidrug efflux transporter MexB
PA4086	*cupB1*	*cupB123456*	1.89	Probable fimbrial subunit CupB1
PA1723	*pscJ*	*pscBCDEFGHIJKL*	1.63	Type III export protein PscJ
PA0958	*oprD*		1.61	Basic amino acid, basic peptide and imipenem outer membrane porin OprD precursor
PA0427	*oprM*	*mexAB-oprM*	1.43	Major intrinsic multiple antibiotic resistance efflux outer membrane protein Opr
PA2491	*mexS*		-2.17	Hypothetical protein (MexEF-OprN regulator)
PA0998	*pqsC*	*pqsABCDE*	-2.83	Homologous to beta-keto-acyl-acyl-carrier protein synthase
**Anaerobic**				
PA1718	*pscE*	*pscBCDEFGHIJKL*	2.23	Type III export protein PscE
PA0958	*oprD*		1.76	Basic amino acid, basic peptide and imipenem outer membrane porin OprD precursor
PA3616		*recA-PA3616*	-1.61	Hypothetical protein
PA3008		*lexA-PA3008*	-1.81	Hypothetical protein
PA3007	*lexA*	*lexA-PA3008*	-1.88	Repressor protein LexA
PA3617	*recA*	*recA-PA3616*	-1.96	RecA protein
PA0807	*ampDh3*		-2.44	Beta-lactamase expression regulator AmpD
PA2485		*PA2485-PA2486*	-2.66	Hypothetical protein
PA4763	*recN*		-2.71	DNA repair protein RecN
PA2486	*ptrC*	*PA2485-PA2486*	-2.94	Hypothetical protein (T3SS regulator)
PA2494	*mexF*	*mexEF-oprN*	-3.01	Resistance-Nodulation-Cell Division (RND) multidrug efflux transporter MexF
PA2484			-3.55	Hypothetical protein

Selected differentially regulated genes, under both aerobic and anaerobic conditions. Complete list of all the genes (>1.5-fold) is available in S2 and S3 Table.

Under anaerobic conditions, despite the hypothetical proteins (PA5507 and PA5509, with log_2_-fold changes of 3.21 and 2.99), *mexS* and *pyoS5* were the more strongly repressed genes (log_2_-fold changes of -4.69 and -3.9) (Table 2 and S3 Table).

Classifying the transcriptionally altered genes in metabolic categories [2] (S4 Fig), the categories in which more genes were included were antibiotic resistance, antibiotic susceptibility and small molecules transportation. By contrast, under anaerobic conditions, the main metabolic category with altered transcription was small molecules transportation.

### NrdR is not essential during *P*. *aeruginosa* infection

We have previously shown that the *nrdJ* and the *nrdD* genes of *P*. *aeruginosa* are important during infection of *Drosophila melanogaster* [7]. As the NrdR regulates the expression of the different *nrd* genes, both aerobically and anaerobically, we wondered whether a mutant for this transcriptional regulatory protein is important during bacterial infections.

**Fig 6:**
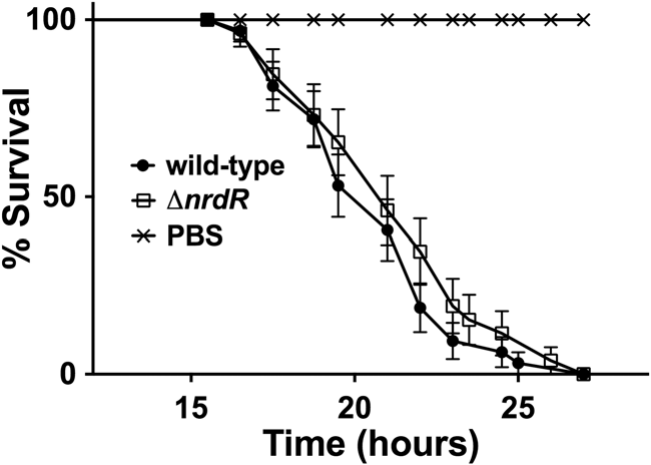
The *nrdR* mutant of *P*. *aeruginosa* does not alter the kinetics of *D*. *melanogaster* killing. Control flies were injected with PBS. Fly survival was monitored for 48 h. Approximately 100 flies were used for each experiment.

Injections of the same number of cells of a wild-type strain and a Δ*nrdR* mutant strain showed exactly the same killing behavior in flies (Fig 6), showing a 50% death rate 25 h post-infection. Therefore, this situation does not alter the virulence capacity of PAO to infect flies despite presenting an up-regulation of all *nrd* genes in the Δ*nrdR* mutant strain.

## Discussion

Ribonucleotide reductases are key enzymes for all living cells, as they are responsible for the dNTP supply that is essential for DNA synthesis and repair. Eukaryotic cells encode only one type of RNR, class Ia, which is responsible for providing the different dNTPs under all conditions. Surprisingly, prokaryotic cells, which can be considered *a priori* as less complex organisms, have the capacity to encode different RNR classes in the same genome [25,26]. The presence of different paralogous genes performing the same enzymatic activity is astonishing, leading us to question why prokaryotes encode different RNR classes. Addressing this question is crucial for the understanding of the transcriptional regulation of each RNR class.

The NrdR factor was first identified in *S*. *coelicolor* [10], and proposed, by phylogenetic profiling, as a potential transcriptional regulator of different RNR genes [11], which was later confirmed in *E*. *coli* [12,13] and mycobacteria [27]. In our study, we aimed to gain insight into the role of this transcriptional factor in *P*. *aeruginosa*; this is the first attempt to study NrdR-related regulation in an organism in which all three RNR classes are encoded [7,28]. The *nrdR* gene also presents a unique genomic context in this bacterium: a polycistronic transcript encoding for *nrdR* and *ribD* genes can be detected (unpublished data), as an evidence of a *nrdR-ribD* operon (Fig 1A), instead of the longer operon that is present in other γ-Proteobacteria (*nrdR-ribD-ribH-nusB*) [11,12].

We showed that NrdR is transcribed under both aerobic and anaerobic conditions, but increases substantially during anaerobic growth, and especially in the exponential growth phase (Fig 1). This increase can be explained through the transcriptional activation by NarL, a transcription factor that is strongly related to anaerobic growth [29], and according to the presented results (Figs 1 and 2, S1 Fig) activates *nrdR* transcription by binding and interacting with two NarL boxes located at the *nrdR* promoter region.

**Fig 7:**
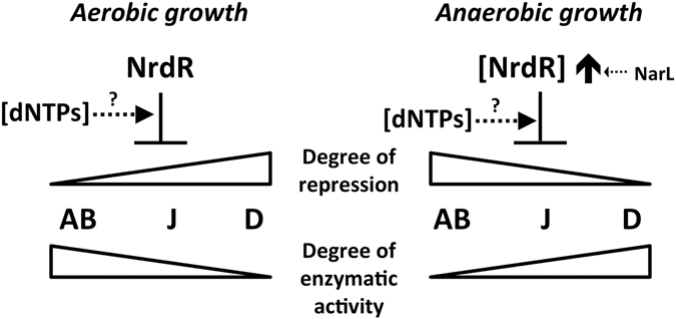
Model of NrdR-related control on RNR gene expression. The degree of repression on each RNR class expression, under aerobic or anaerobic conditions, is opposite to the enzymatic activity of these classes under each condition. Considering the presence of an ATP-cone domain in NrdR, dNTPs level alterations could also be affecting the results.

As expected, NrdR regulates all three RNR classes, but surprisingly, it acts differently during aerobic or anaerobic growth. Aerobically, NrdR acts as a repressor of all RNR genes (Fig 3), although maximum repression is exerted on class II and class III RNRs, while class Ia repression is less strict, conforming fully with the hypothesis that class Ia supports aerobic growth in this bacterium [3,7,8,28,30]. By contrast, NrdR does not repress class II and class III expression under anaerobic conditions, showing only a slight repression of class Ia RNR: as class II and III RNRs support the *P*. *aeruginosa* anaerobic growth [7]; this also fits with our model (Fig 7). The results of the gene reporter assay were confirmed by qRT-PCR (Fig 3), therefore providing strong evidence supporting our model.

Two NrdR boxes have been identified in each RNR gene promoter (S2 and S3 Figs). Although we could not obtain pure soluble NrdR to perform DNA gel shift assays (all attempts to overproduce this protein lead to the formation of inclusion bodies), the results of the gene reporter assay with a mutation in the identified NrdR boxes agree with our previous results [12]; in all cases we have mutated the NrdR-box2 (because NrdR-box1 overlaps with the -10 promoter sequence), and we have obtained similar expression levels compared with the Δ*nrdR* mutant strain demonstrating the physiological role of these DNA regions to bind a functional NrdR.

To this point we can assume that we have strong evidence to support our working model (Fig 7); RNR activity in *P*. *aeruginosa* is controlled by the NrdR factor, which acts by binding in the NrdR boxes in all RNR gene promoters (it is believed that it can act by forming a dimer to bind the two characteristic boxes), and repressing RNR activity according to the needs of the bacterium: high repression of class II and III RNRs under aerobic conditions, and repression of class I only under anaerobic conditions.

As the NrdR protein harbors an ATP-cone domain that is able to bind nucleotides, it seems logical to assume that NrdR activity is modulated by differential nucleotide binding [13,14] so that high dNTP levels (indicating high RNR activity) might activate NrdR-related repression. To do so, its ATP-cone is likely to be fully occupied (to its allosterically controlled limit) with ATP in usual situations, but competent to bind dATP and act as a dNTP pool sensor [14]; the binding of the nucleotide should control the oligomeric state of NrdR by a conformational change in the zinc-finger domain, and thereby modulate its interaction with the NrdR boxes. To evaluate this control we compared RNR transcription in wild-type and Δ*nrdR* mutant strains while treating with hydroxyurea (decreasing dATP levels, and hence modulating the bound nucleotide) but we did not obtain significant results (data not shown), most likely because treatment with hydroxyurea only affected class Ia RNR activity (and not class II and class III RNRs), and therefore the dNTP pool slightly decreased (Fig 3) and class II was fully active [7]. In other studies in *S*. *typhimurium* and *Chlamydia sp* [23,24], hydroxyurea treatment completely abolished the dNTP supply, making this type of study far easier. In our model the role of the alteration of the dNTP pool in the fine-tuning transcriptional regulation of the different RNR genes by NrdR is still inconclusive. Despite the lack of data in our working model, which was still not completely set, we hypothesized that NrdR could be responding to alteration of the dNTP pool, inhibiting RNR gene transcription when necessary. In contrast to anaerobic conditions when only class Ia RNR is affected by NrdR, during aerobic growth NrdR is able to down-regulate all RNR gene transcription, so this response to increased dNTPs may be its main role under this condition. This model is summarized in Fig 7.

Moreover, we also identified for the first time that NrdR is not only involved in RNR activity regulation but also regulates *topA* expression, a gene encoding for *P*. *aeruginosa* topoisomerase I. The *topA* promoter region presents only a single NrdR putative binding site (S2 Fig), suggesting different NrdR binding and regulation on this gene compared to RNR genes. In agreement with this hypothesis, we have shown that NrdR up-regulates *topA* transcription, instead of repressing it (Fig 4). As with RNR activity, we confirmed this effect at a physiological level: as TopA relaxes negative supercoiled DNA, a high level of negative supercoiled DNA appears in a Δ*nrdR* mutant strain, during exponential growing phase, without NrdR-related topA activation (Fig 4B).

There were no more promoters harboring putative NrdR boxes, but, according to our global transcriptomic analysis results, in a Δ*nrdR* mutant strain, a small but significant group of 47 genes (log_2_-fold change>1.5) was deregulated during aerobic conditions, and 111 genes (log_2_-fold change>1.5) presented similar behavior during anaerobic conditions (Fig 6, S2 and S3 Tables). Among those genes we can identify some related to the SOS system, antibiotic resistance, transport of small molecules, etc (S4 Fig). This deregulation does not lead to a loss of infectivity (Fig 6). Given the absence of putative NrdR-boxes on the promoter regions of the deregulated genes detected in the array, we believe the change in the expression of these genes to be due to indirect effects. We propose that TopA down-regulation in the absence of NrdR may alter gene transcription by changing DNA topology and causing the accumulation of cleavage complexes. For instance, it has been described that SOS system can be deregulated by TopA depletion during antibiotic treatment [31]. In addition, some of the deregulated genes appear to also show altered transcription in a Δ*topA* mutant strain, according to a previous transcriptomic assay by the Lawrence G. Rahme group (unpublished data, Gene Expression Omnibus GSE24038).

The difference observed between the two groups of deregulated genes under aerobic and anaerobic conditions provides further evidence of the differential behavior of NrdR in *P*. *aeruginosa* as we have proposed, although the NarL-related activation and the dNTP-binding modulation may not be the only systems acting on this regulation.

In summary, this study has provided evidence of control of the three RNR operons and the *topA* gene by NrdR in *P*. *aeruginosa*, which is a differential control sensitive to oxygenation conditions and the growth phase. This control clearly plays an important role in the coordination of the expression of the different RNRs, dictating which RNR is expressed under certain growing conditions. By studying this and other factors controlling RNR activity we will be nearer to an explanation of the apparent redundancy among the three RNR classes, and to an understanding of how this bacterium uses all three classes to survive under different environmental conditions.

## Supporting Information

S1 Fig NarL-dependent expression of *nrdR*. Fluorescence intensity measurements of *P*. *aeruginosa nrdR* promoter fusions compared with their mutagenized NarL boxes (box1 and box2, three different mutations in each one), expressed in relative fluorescence units. The experiment was performed in a wild-type *P*. *aeruginosa* background (pETS161 (wt), pETS181 (box1), pETS182 (box1.2), pETS183 (box1.3), pETS184 (box2), pETS185 (box2.2), pETS186 (box2.3) and pEST187 (box1 and 2)) and in a Δ*narL* background (only wt promoter, pETS161). Strains were grown anaerobically until the mid-logarithmic phase. Values represent the mean of three independent experiments. *: Significantly different compared with wild-type promoter region (pETS161) in an unpaired *t*-test (*P*<0.05).
(TIFF)

S2 Fig NrdR binding sites in the three *P*. *aeruginosa* PAO1 RNRs and *topA* promoter regions. An overview of the entire operon is shown, with open rectangular frames indicating the two NrdR boxes in RNR promoters and the one in the *topA* promoter. The detailed sequence of the area surrounding the boxes is displayed below; the predicted NrdR binding sites are indicated by the nucleotides in bold and black boxes. The position of the NrdR boxes is given relative to the translation start codon of the first gene of the *nrd* operon, as previously described (Rodionov DA and Gelfand MS (2005) Identification of a bacterial regulatory system for ribonucleotide reductases by phylogenetic profiling. Trends in Genetics 21:385–389).
(TIF)

S3 Fig Site-directed mutagenesis of the predicted NrdR box in RNR promoters and *topA* promoter. Representation of RNR and *topA* promoters’ region sequence of *P*. *aeruginosa* strain PAO1 indicating the NrdR binding sites. Black boxes indicate NrdR recognition sites, and the NrdR box2 mutated residues are shown in upper case and in bold letters. Fluorescence measurements of *P*. *aeruginosa* RNR promoter fusions (pETS134, pETS180 and pETS136) and P*topA* (pETS177) compared with their mutagenized NrdR mutated box2 (pETS188, pETS189, pETS190 and pETS178, respectively) were measured in relative fluorescence units (RFUs) in a wild-type *P*. *aeruginosa* background and in a Δ*nrdR* background. Strains were grown aerobically and anaerobically until the mid-logarithmic phase. Values represent the mean of three independent experiments. *: Significantly different compared with wild-type promoter region (pETS161) in an unpaired *t*-test (*P*<0.05).
(TIF)

S4 Fig Distribution of deregulated genes in the global transcriptional analysis according to assigned metabolic classes [2]. (TIFF)

S5 Fig Transcriptional analysis of deregulated genes on global transcriptional analysis of a Δ*nrdR* mutant strain. Total RNA was reverse transcribed with gene-specific primers as described in Materials and Methods. The analysis demonstrates the specificity of global transcriptional analysis in the absence of *nrdR*. *gapA* was used as internal standard.
(TIF)

S1 Table Primers and probes used in this study. (PDF)

S2 Table Global transcriptomic analysis of a Δ*nrdR* mutant strain compared with *P*. *aeruginosa* PAO1 wild-type strain grown aerobically. (PDF)

S3 Table Global transcriptomic analysis of a Δ*nrdR* mutant strain compared with *P*. *aeruginosa* PAO1 wild-type strain grown anaerobically. (PDF)
