# Supplementary figures and images for: Function of the Pseudomonas aeruginosa NrdR Transcription Factor: Global Transcriptomic Analysis and Its Role on Ribonucleotide Reductase Gene Expression
Source: PLoS One. 2015 Apr 24;10(4):e0123571. doi: 10.1371/journal.pone.0123571 (PMC4409342; doi:10.1371/journal.pone.0123571)

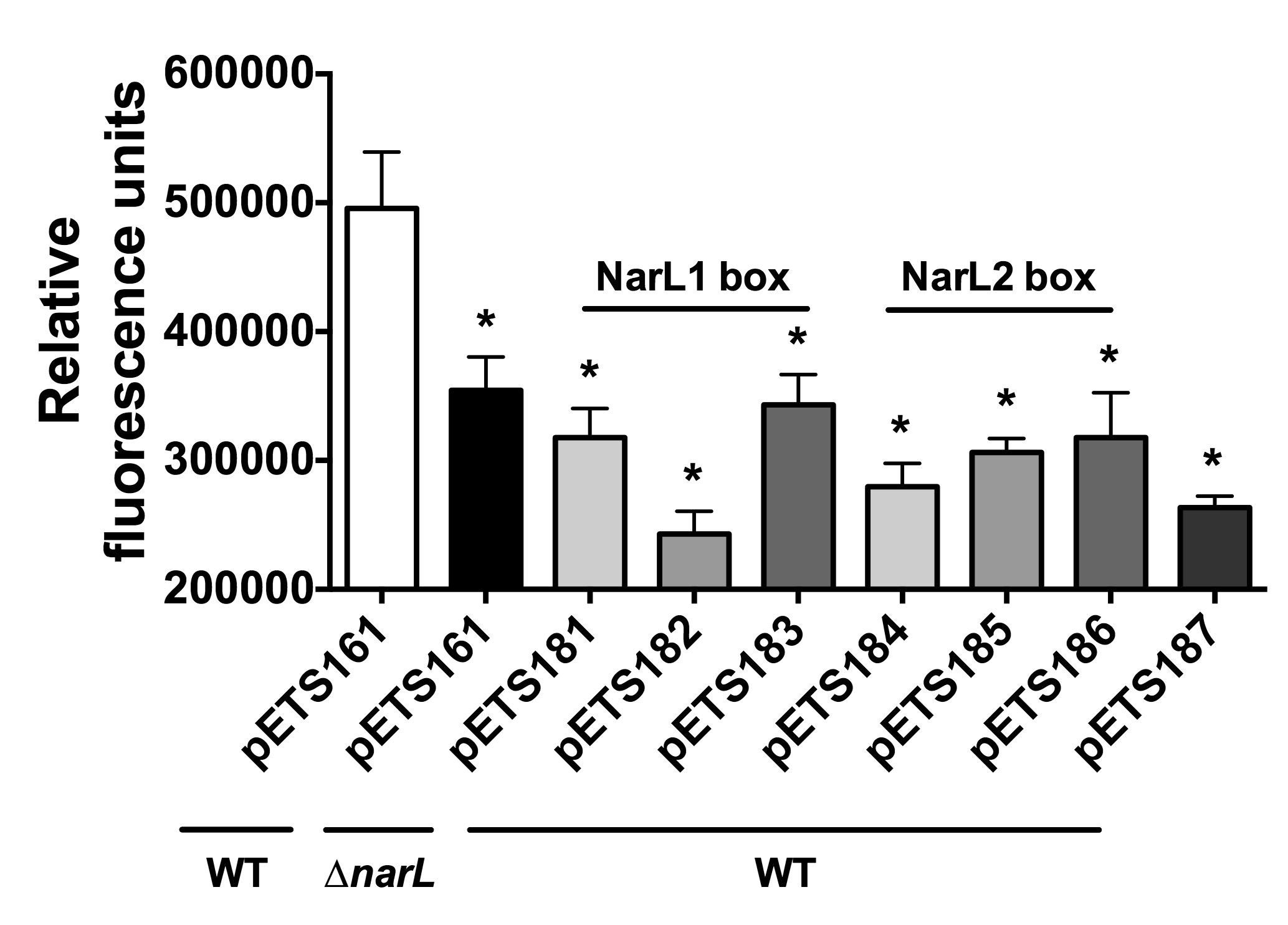

Supplement: S1 Fig — Fluorescence intensity measurements of P. aeruginosa nrdR promoter fusions compared with their mutagenized NarL boxes (box1 and box2, three different mutations in each one), expressed in relative fluorescence units. The experiment was performed in a wild-type P. aeruginosa background (pETS161 (wt), pETS181 (box1), pETS182 (box1.2), pETS183 (box1.3), pETS184 (box2), pETS185 (box2.2), pETS186 (box2.3) and pEST187 (box1 and 2)) and in a ΔnarL background (only wt promoter, pETS161). Strains were grown anaerobically until the mid-logarithmic phase. Values represent the mean of three independent experiments. *: Significantly different compared with wild-type promoter region (pETS161) in an unpaired t-test (P<0.05). (TIFF) [file pone.0123571.s001.tiff]

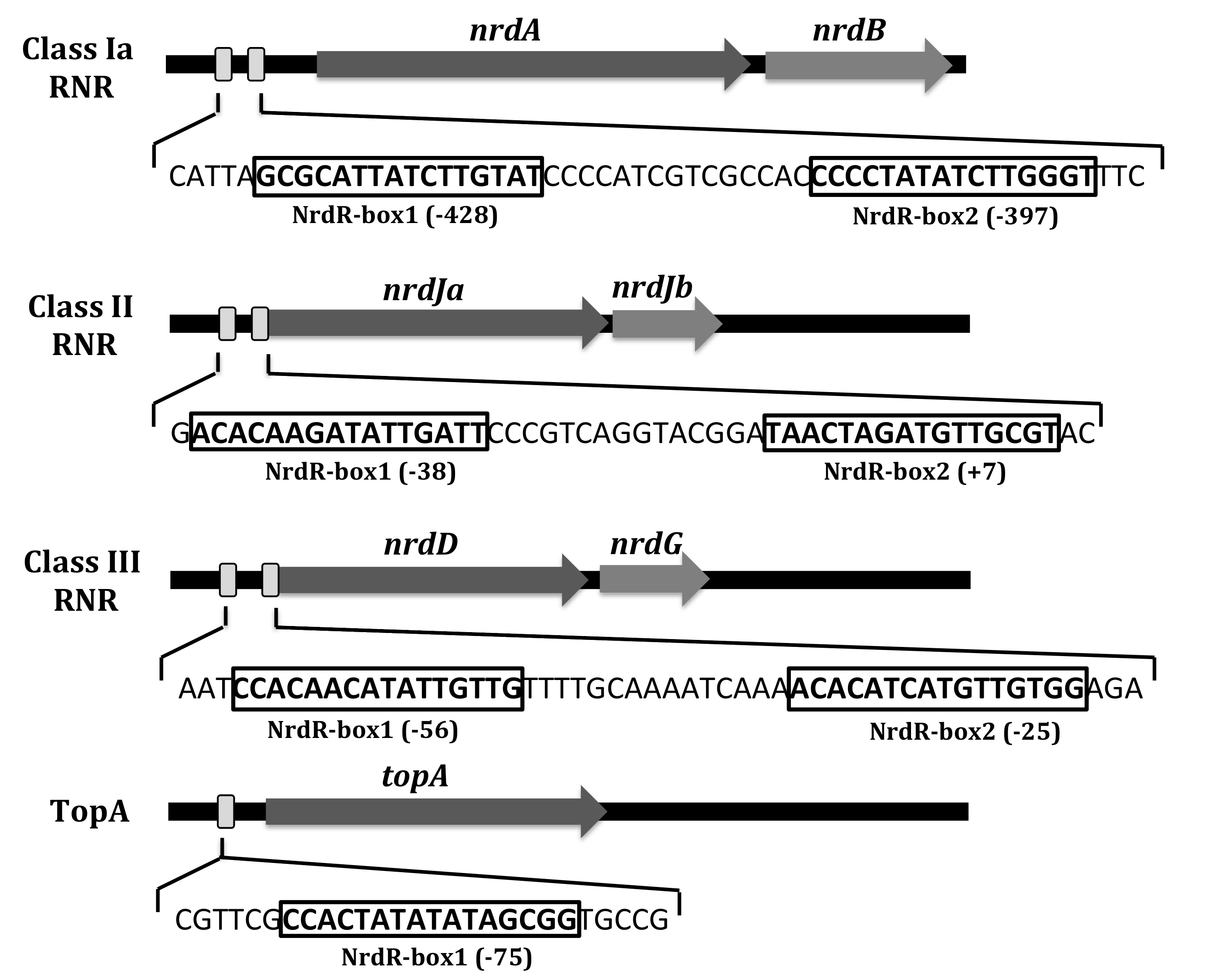

Supplement: S2 Fig — An overview of the entire operon is shown, with open rectangular frames indicating the two NrdR boxes in RNR promoters and the one in the topA promoter. The detailed sequence of the area surrounding the boxes is displayed below; the predicted NrdR binding sites are indicated by the nucleotides in bold and black boxes. The position of the NrdR boxes is given relative to the translation start codon of the first gene of the nrd operon, as previously described (Rodionov DA and Gelfand MS (2005) Identification of a bacterial regulatory system for ribonucleotide reductases by phylogenetic profiling. Trends in Genetics 21:385–389). (TIF) [file pone.0123571.s002.tif]

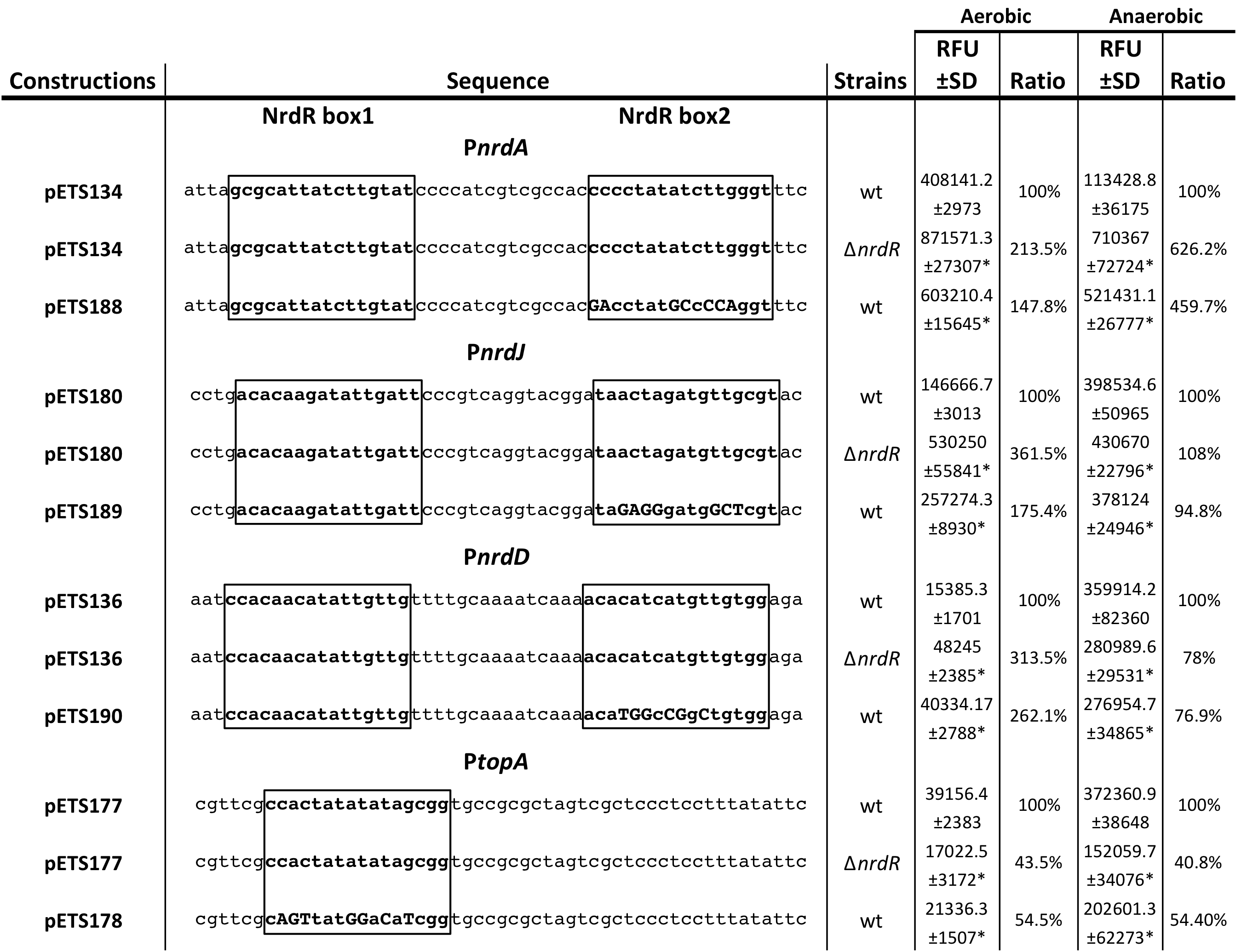

Supplement: S3 Fig — Representation of RNR and topA promoters’ region sequence of P. aeruginosa strain PAO1 indicating the NrdR binding sites. Black boxes indicate NrdR recognition sites, and the NrdR box2 mutated residues are shown in upper case and in bold letters. Fluorescence measurements of P. aeruginosa RNR promoter fusions (pETS134, pETS180 and pETS136) and PtopA (pETS177) compared with their mutagenized NrdR mutated box2 (pETS188, pETS189, pETS190 and pETS178, respectively) were measured in relative fluorescence units (RFUs) in a wild-type P. aeruginosa background and in a ΔnrdR background. Strains were grown aerobically and anaerobically until the mid-logarithmic phase. Values represent the mean of three independent experiments. *: Significantly different compared with wild-type promoter region (pETS161) in an unpaired t-test (P<0.05). (TIF) [file pone.0123571.s003.tif]

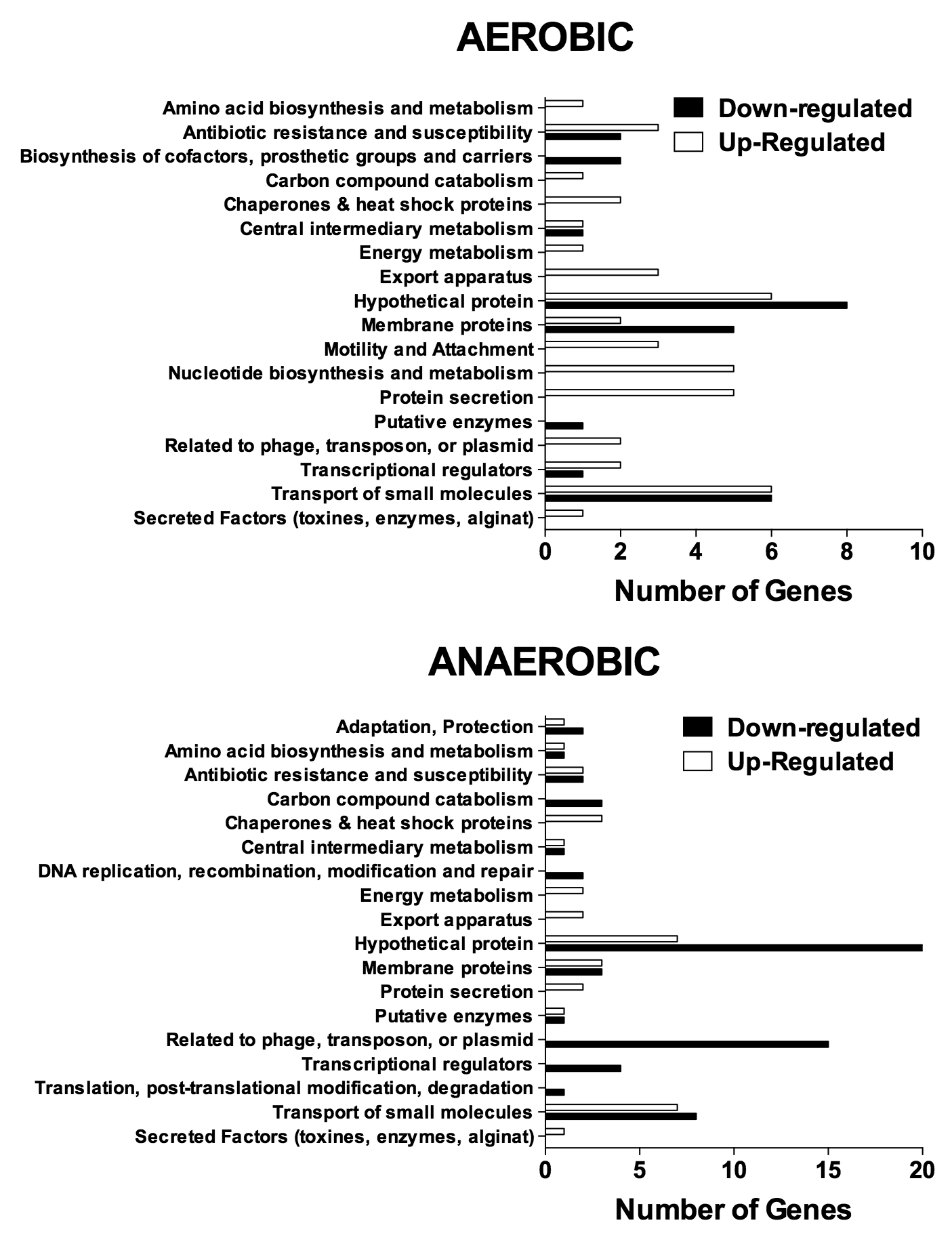

Supplement: S4 Fig — (TIFF) [file pone.0123571.s004.tiff]

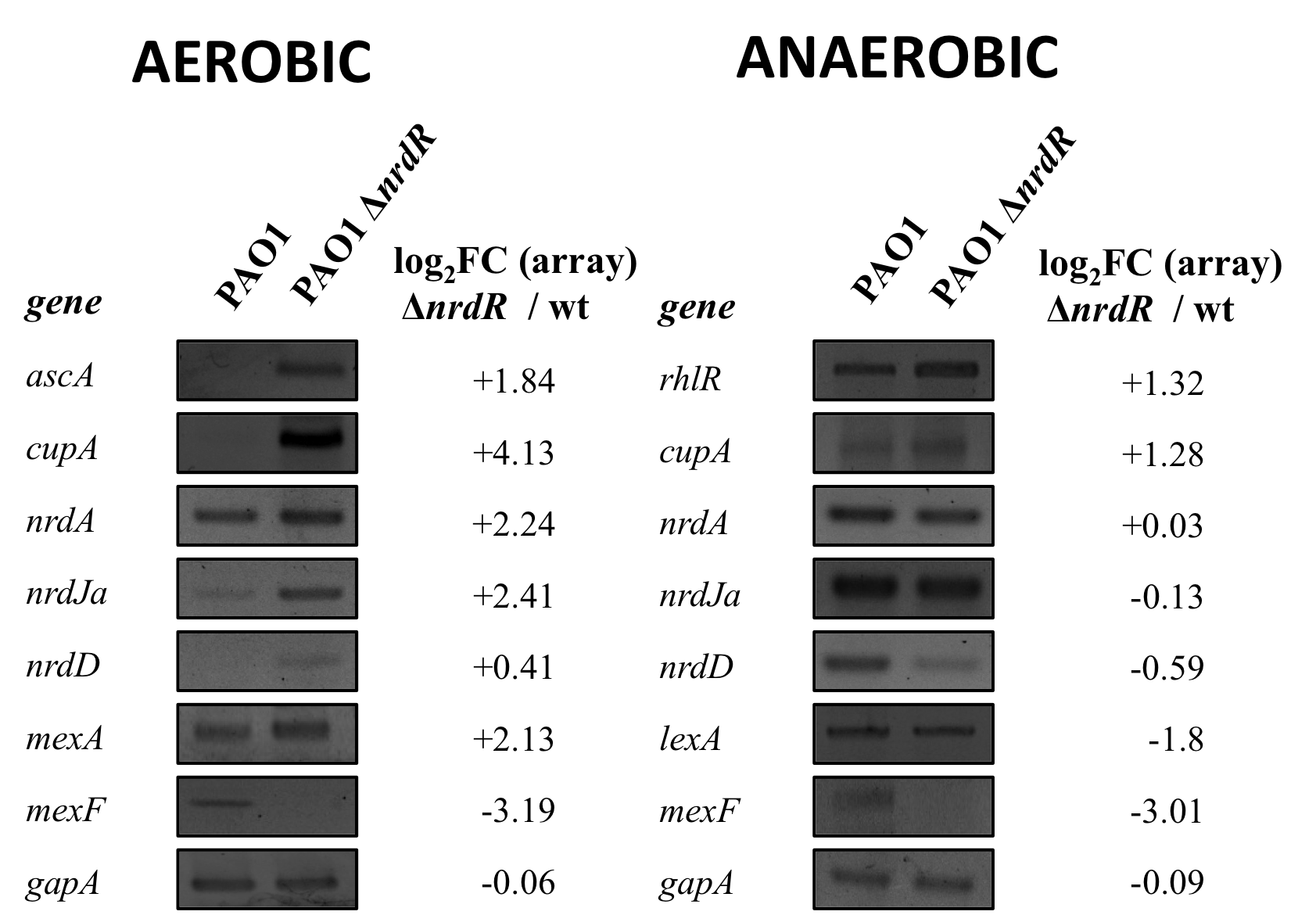

Supplement: S5 Fig — Total RNA was reverse transcribed with gene-specific primers as described in Materials and Methods. The analysis demonstrates the specificity of global transcriptional analysis in the absence of nrdR. gapA was used as internal standard. (TIF) [file pone.0123571.s005.tif]
