# Supplementary material for: Function of the Pseudomonas aeruginosa NrdR Transcription Factor: Global Transcriptomic Analysis and Its Role on Ribonucleotide Reductase Gene Expression
Source: PLoS One. 2015 Apr 24;10(4):e0123571. doi: 10.1371/journal.pone.0123571 (PMC4409342; doi:10.1371/journal.pone.0123571)
Supplement: S1 Table — (PDF) [file pone.0123571.s006.pdf]

S1 Table. **Primers and probes used in this study.**

Name	Sequence (5'→3')	Application
PnrdRBHI-up	AGGATCCAGGAGAAGGACGGCCAGCAG	Promoter-probe cloning
PnrdRClaI-lw	AAAATCGATATGGTGTCATGGGCACCGCAG	Promoter-probe cloning
NrdRHindIII-low	AAGCTTCATGTACAGCGGGT	Cloning
pBBR1-up	CATCGCAGTCGGCCTATTGG	Check-Cloning
pBBR1-lw	CACTTTATGCTTCCGGCTCG	Check-Cloning
M13-dir	GTTTTCCCAGTCACGAC	Check-Cloning
M13-rev	CAGGAAACAGCTATGAC	Check-Cloning
pUCP20T-up	CCTCTTCGCTATTACGCCAG	Cloning
pUCP20T-low	TCCGGCTCGTATGTTGTGTG	Cloning
PtopA BamHI-up	GGATCCGAAGAGGCGCTGGTGATCTA	Cloning
PtopA ClaI-low	ATCGATGTACTGGCTGCCCAGGTACT	Cloning
PnrdA-up	AGGATCCGAATTCTTGCTCCACACAGCCTC	Cloning/RT-PCR
PnrdA-low	ACCCGGGTTCTCGCGTGTGGTGTCG	Cloning/RT-PCR
PnrdJ BamHI new-up	GGATCCCGCGCCCAGCTGAAGGCC	Cloning/RT-PCR
PnrdJ SmaI new-low	AACCCGGGGACTGCGTTGCGTCTGTC	Cloning/RT-PCR
PnrdD-up	AGGATCCGAATTCGCCCGCCTCGCCCAGG	Cloning/RT-PCR
PnrdD new-low	AATCGATCAGGGTGGCCGGCCAGGTAG	Cloning/RT-PCR
AmR2-up	CAACGACCTATGCCCCAGGTT	Mutation of NrdRbox2 in P*nrdA*
AmR2-low	AACCTGGGGCATAGGTCGTTG	Mutation of NrdRbox2 in P*nrdA*
JmR2-up	GGATAGAGGGATGGCTCGTAC	Mutation of NrdRbox2 in P*nrdJ*
JmR2-low	GTACGAGCCATCCCTCTATCC	Mutation of NrdRbox2 in P*nrdJ*
DmR2-up	AAACATGGCCGGCTGTGGAG	Mutation of NrdRbox2 in P*nrdD*
DmR2-low	CTCCACAGCCGGCCATGTTT	Mutation of NrdRbox2 in P*nrdD*
TmR up	CAGTTATGGACATCGGTGCC	Mutation of NrdRbox2 in P*topA*
TmR low	GGCACCGATGTCCATAACTG	Mutation of NrdRbox2 in P*topA*
nrdATaqM-up	CCCTTCCTGAAAGTGGTCAA	qRT-PCR
nrdATaqM2-low	TGTTCATGTCGTGGGTACG	qRT-PCR
nrdJTaqM-up	CGGGTCAACGAACTGAACA	qRT-PCR
nrdJTaqM2-low	GTAAACACCCGCACCACTTC	qRT-PCR
nrdDTaqM-up	CCGAGATGGACCTGATCAAC	qRT-PCR
nrdDTaqM2-low	CCGAGTTGAGGAAGTTCTGG	qRT-PCR

nrdRTaqM-up	GTTCGACGAGGACAAGCTG	RT-PCR
nrdRTaqM2-low	ATGTAGGCGACTTCGTCGAG	RT-PCR
gapTaqM-up	GAGTGCACGGGGCTCTTC	qRT-PCR
gapTaqM-low	GAGGTTCTGGTCGTTGGT	qRT-PCR
mexA-up	GCCATGCGTGTACTGGTTCC	RT-PCR
mexA-low	GCTCTGGTAGTCGGCCTCGT	RT-PCR
mexF-up	CGAACTACGCGGTGCTCAAC	RT-PCR
mexF-low	GCGCGGATGATGATGTTCTC	RT-PCR
cupA-up	GTGATCCTCGACAGCGTACC	RT-PCR
cupA-low	GTCGTGCTGGTGCTGGTG	RT-PCR
RhlRNdeI-up	ACATATGAGGAATGACGGAGGCT	RT-PCR
RhlR-low	ATCAGATGAGACCCAGCGC	RT-PCR
acsAa up	TGGTACGACGACCTGATGAA	RT-PCR
acsAa low	CCTCGAACAGAATGGTGGTG	RT-PCR
PAlexA- up	ACATATGCAGAAGCTGACGCC	RT-PCR
PAlexA-lw	ACTCGAGTCAGCGCCGGATCACG	RT-PCR
nrdA-FAM	CTGGCACCTGGACATC	qRT-PCR probe
nrdJ-FAM	TCGGCTCGGTCAACCT	qRT-PCR probe
nrdD-FAM	CCCGACCTACAACATC	qRT-PCR probe
gap-FAM	CCTGCACCACCAACTG	qRT-PCR probe
mutNarL1 up	CGCGGATGCGCCCAATTGCGGCTTCCTTCA	Mutation of NarLbox1 in P*nrdR*
mut NarL1 low	TGAAGGAAGCCGCAATTGGGCGCATCCGCG	Mutation of NarLbox1 in P*nrdR*
mutNarL1.2 up	CGCGGATGCGCTGGCATGCGGCTTCCTTCA	Mutation of NarLbox1 in P*nrdR*
mutNarL1.2 low	TGAAGGAAGCCGCATGCCAGCGCATCCGCG	Mutation of NarLbox1 in P*nrdR*
mutNarL1.3 up	CGCGGATGCGCTGCCATGCGGCTTCCTTCA	Mutation of NarLbox1 in P*nrdR*
mutNarL1.3 low	TGAAGGAAGCCGCATGGCAGCGCATCCGCG	Mutation of NarLbox2 in P*nrdR*
mutNarL R dir	TTCCTTCAGCATAATTAGTATCAGCACCA	Mutation of NarLbox2 in P*nrdR*
mutNarL R rev	TGGTGCTGATACTAATTATGCTGAAGGAA	Mutation of NarLbox2 in P*nrdR*
mut NarL2.2 up	TTCCTTCAGCTGGGCCTGTATCAGCACCA	Mutation of NarLbox2 in P*nrdR*
mut NarL2.2 low	TGGTGCTGATACAGGCCCAGCTGAAGGAA	Mutation of NarLbox2 in P*nrdR*
mut Narl2.3 up	TTCCTTCAGCTGCGCCTGTATCAGCACCA	Mutation of NarLbox2 in P*nrdR*
mutNarL 2.3 low	TGGTGCTGATACAGGCGCAGCTGAAGGAA	Mutation of NarLbox2 in P*nrdR*
