# Supplementary material for: Function of the Pseudomonas aeruginosa NrdR Transcription Factor: Global Transcriptomic Analysis and Its Role on Ribonucleotide Reductase Gene Expression
Source: PLoS One. 2015 Apr 24;10(4):e0123571. doi: 10.1371/journal.pone.0123571 (PMC4409342; doi:10.1371/journal.pone.0123571)
Supplement: S2 Table — (PDF) [file pone.0123571.s007.pdf]

S2 Table: Global transcriptomic analysis of a Δ*nrdR* mutant strain compared with *P. aeruginosa* PAO1 wild-type strain grown aerobically. List of all differentially regulated genes identified (more than 1.5-fold change in expression).

ID	*Gene*	*Operon arrangement*	Log2 Fold Change	*Gene Product*
PA2128	*cupA1*	*cupA12345*	4.13	Fimbrial subunit CupA1
PA4139			3.78	Hypothetical protein
PA1383			2.42	Hypothetical protein
PA5497	*nrdJa*	*nrdJab*	2.41	Class II (cobalamin-dependent) ribonucleotide-diphosphate reductase subunit, NrdJa
PA2223			2.37	Hypothetical protein
PA1718	*pscE*	*pscBCDEFGHIJKL*	2.32	Type III export protein PscE
PA1156	*nrdA*	*nrdAB*	2.24	Ribonucleoside reductase, large chain
PA0992	*cupC1*	*cupC123*	2.19	Fimbrial subunit CupC1
PA0425	*mexA*	*mexAB-oprM*	2.13	Resistance-Nodulation-Cell Division (RND) multidrug efflux membrane fusion protein
PA3188			2.11	Hypothetical protein
PA0424	*mexR*		2.06	Multidrug resistance operon repressor MexR
PA1693	*pscR*	*PA1697-pscOPQRSTU*	2.00	Translocation protein in type III secretion
PA3144			1.99	Hypothetical protein
PA2322			1.98	Hypothetical protein
PA3842			1.96	Hypothetical protein

PA1155	*nrdB*	*nrdAB*	1.96	Ribonucleoside reductase, small chain
PA0865	*hpd*		1.94	4-hydroxyphenylpyruvate dioxygenase
PA0426	*mexB*	*mexAB-oprM*	1.93	Resistance-Nodulation-Cell Division (RND) multidrug efflux transporter MexB
PA4086	*cupB1*	*cupB123456*	1.89	Probable fimbrial subunit CupB1
PA0887	*acsA*		1.84	Acetyl-coenzyme A synthetase
PA0978			1.78	Hypothetical protein
PA5169			1.77	Hypothetical protein
PA1333			1.65	Hypothetical protein
PA1723	*pscJ*	*pscBCDEFGHIJKL*	1.63	Type III export protein PscJ
PA0958	*oprD*		1.61	Basic amino acid, basic peptide and imipenem outer membrane porin OprD precursor
PA3720			1.61	Hypothetical protein
PA1386			1.54	Hypothetical protein
PA5491			1.53	Hypothetical protein
PA0717			1.51	Hypothetical protein
PA0427	*oprM*	*mexAB-oprM*	1.43	Major intrinsic multiple antibiotic resistance efflux outer membrane protein Opr
PA2813			-1.58	Hypothetical protein
PA4881			-1.59	Hypothetical protein
PA0567			-1.64	Hypothetical protein
PA2812			-1.68	Hypothetical protein
PA2811			-1.75	Hypothetical protein

PA0281	*cysW*		-2.05	Sulfate transport protein CysW
PA2491	*mexS*		-2.18	Hypothetical protein
PA3931			-2.21	Hypothetical protein
PA3229			-2.37	Hypothetical protein
PA4691			-2.55	Hypothetical protein
PA0998	*pqsC*	*pqsABCDE*	-2.83	Homologous to beta-keto-acyl-acyl-carrier protein synthase
PA0565			-3.06	Hypothetical protein
PA2494	*mexF*		-3.19	Resistance-Nodulation-Cell Division (RND) multidrug efflux transporter MexF
PA3281			-3.73	Hypothetical protein
PA3283			-4.57	Hypothetical protein
