# Supplementary material for: Function of the Pseudomonas aeruginosa NrdR Transcription Factor: Global Transcriptomic Analysis and Its Role on Ribonucleotide Reductase Gene Expression
Source: PLoS One. 2015 Apr 24;10(4):e0123571. doi: 10.1371/journal.pone.0123571 (PMC4409342; doi:10.1371/journal.pone.0123571)
Supplement: S3 Table — (PDF) [file pone.0123571.s008.pdf]

**S3 Table: Global transcriptomic analysis of a Δ*nrdR* mutant strain compared with *P. aeruginosa* PAO1 wild-type strain grown anaerobically.** List of all differentially regulated genes identified (more than 1.5-fold change in expression).

| ID | *Gene* | *Operon arrangement* | Log2 Fold Change | *Gene Product* |
|---|---|---|---|---|
| PA5507 | | *PA5506-PA5507-pauA7-PA5509* | 3.2 | Hypothetical protein |
| PA5509 | | *PA5506-PA5507-pauA7-PA5509* | 2.98 | Hypothetical protein |
| PA4058 | | *PA4058-PA4059-PA4060* | 2.23 | Hypothetical protein |
| PA1718 | *pscE* | *exsD-pscBCDEFGHIJKL* | 2.23 | Type III export protein PscE |
| PA1984 | *exaC* | *exaB-exaC* | 2.13 | Hypothetical protein |
| PA0713 | | | 2.03 | Hypothetical protein |
| PA1556 | *ccoO2* | *ccoN2-ccoO2* | 1.90 | Hypothetical protein |
| PA1073 | *braD* | *braD-braE-braF-braG* | 1.79 | Branched-chain amino acid transport protein BraD |
| PA1337 | *ansB* | *ansB-PA1336-PA1335* | 1.77 | Glutaminase-asparaginase |
| PA1340 | | | 1.76 | Hypothetical protein |
| PA1555 | *ccoP2* | | 1.75 | Hypothetical protein |
| PA0958 | *oprD* | | 1.7 | Basic amino acid, basic peptide and imipenem outer membrane porin OprD precursor |
| PA1072 | *braE* | *braD-braE-braF-braG* | 1.69 | Branched-chain amino acid transport protein BraE |
| PA1070 | *braG* | *braD-braE-braF-braG* | 1.68 | Branched-chain amino acid transport protein BraG |
| PA5506 | | *PA5506-PA5507-pauA7-PA5509* | 1.67 | Hypothetical protein |

| | | | | |
|---|---|---|---|---|
| PA1341 | | *PA1341-PA1340-PA1339* | 1.63 | Hypothetical protein |
| PA2436 | | *PA2436-PA2435-PA2434* | 1.62 | Hypothetical protein |
| PA1596 | *htpG* | *htpG-PA1597* | 1.57 | Heat shock protein HtpG |
| PA3842 | *spcS* | *spcS-PA3843* | 1.56 | Hypothetical protein |
| PA1339 | | *PA1341-PA1340-PA1339* | 1.54 | Hypothetical protein |
| PA4587 | *ccpR* | | 1.54 | Cytochrome c551 peroxidase precursor |
| PA1338 | *ggt* | | 1.53 | Gamma-glutamyltranspeptidase precursor |
| PA1571 | | | 1.53 | Hypothetical protein |
| PA0026 | *plcB* | *plcB-PA0027-PA0028* | -1.50 | Phospholipase C, PlcB |
| PA5107 | *blc* | *fbp-PA5109-PA5108-blc* | -1.50 | Outer membrane lipoprotein Blc |
| PA0612 | *ptrB* | *ptrB-PA0613* | -1.54 | Transcriptional regulators |
| PA0909 | | *PA0908-PA0909* | -1.54 | Membrane proteins |
| PA0462 | | *PA0462-creB-creC* | -1.55 | Hypothetical, unclassified, unknown |
| PA3720 | | *PA3720-armR* | -1.55 | Hypothetical, unclassified, unknow |
| PA5526 | | | -1.55 | Hypothetical, unclassified, unknown |
| PA4817 | | *PA4816-PA4817* | -1.56 | Hypothetical, unclassified, unknown |
| PA5217 | | *PA5218-PA5217-PA5216* | -1.58 | Transport of small molecules |
| PA0490 | | *PA0491-PA0490* | -1.59 | Hypothetical, unclassified, unknown |
| PA5212 | | | -1.60 | Hypothetical, unclassified, unknown |
| PA3616 | | *recA-PA3616* | -1.61 | Hypothetical, unclassified, unknown |

| | | | | |
|---|---|---|---|---|
| PA0284 | | | -1.68 | Hypothetical, unclassified, unknown |
| PA0327 | | | -1.69 | Hypothetical, unclassified, unknown |
| PA4515 | | *PA4515-PA4516* | -1.72 | Hypothetical, unclassified, unknown |
| PA3413 | | *PA3413-PA3414* | -1.73 | Hypothetical, unclassified, unknown |
| PA0529 | | | -1.79 | Hypothetical, unclassified, unknown |
| PA3008 | | *lexA-PA3008* | -1.81 | Hypothetical, unclassified, unknown |
| PA0130 | *bauC* | *bauA-bauB-bauC* | -1.83 | Carbon compound catabolism |
| PA3007 | *lexA* | *lexA-PA3008* | -1.88 | Repressor protein LexA |
| PA0922 | | | -1.89 | Hypothetical, unclassified, unknown |
| PA3268 | | | -1.91 | Membrane proteins |
| PA0132 | *bauA* | *bauA-bauB-bauC* | -1.92 | Amino acid biosynthesis and metabolism |
| PA0132 | *recA* | *recA-PA3616* | -1.96 | RecA protein |
| PA0131 | *bauB* | *bauA-bauB-bauC* | -2.01 | Carbon compound catabolism |
| PA0283 | *sbp* | | -2.01 | Sulfate-binding protein precursor |
| PA0204 | | *PA0206-PA0205-PA0204-PA0203* | -2.17 | Membrane proteins/Transport of small molecules |
| PA0622 | | *PA0622-PA0623-PA0624* | -2.18 | Related to phage, transposon, or plasmid |
| PA2813 | | *PA2813-PA2812-PA2811* | -2.24 | Central intermediary metabolism |
| PA0911 | | | -2.32 | Hypothetical, unclassified, unknown |
| PA4195 | | *PA4195-PA4194-PA4193-PA4192-PA4191* | -2.33 | Transport of small molecules |
| PA3445 | | | -2.34 | Hypothetical, unclassified, unknown |

| | | | | |
|---|---|---|---|---|
| PA0621 | | *PA0617-PA0618-PA0619-PA0620-PA0621* | -2.4 | Related to phage, transposon, or plasmid |
| PA5525 | | *PA5524-PA5525* | -2.42 | Transcriptional regulators |
| PA0641 | | | -2.44 | Related to phage, transposon, or plasmid |
| PA0807 | *ampDh3* | | -2.44 | Antibiotic resistance and susceptibility |
| PA3866 | | | -2.45 | Secreted Factors (toxins, enzymes, alginate) |
| PA0646 | | *PA0646-PA0647-PA0648* | -2.48 | Hypothetical, unclassified, unknown |
| PA3931 | | | -2.48 | Hypothetical, unclassified, unknown |
| PA0610 | *prtN* | | -2.49 | Transcriptional regulator PrtN |
| PA0633 | | *PA0633-PA0634-PA0635* | -2.52 | Related to phage, transposon, or plasmid |
| PA0203 | | *PA0206-PA0205-PA0204-PA0203* | -2.59 | Transport of small molecules |
| PA1150 | *pys2* | | -2.62 | Pyocin S2 |
| PA0642 | | | -2.63 | Related to phage, transposon, or plasmid |
| PA0648 | | *PA0646-PA0647-PA0648* | -2.63 | Related to phage, transposon, or plasmid |
| PA0615 | | *PA0614-PA0615-PA0616* | -2.67 | Hypothetical, unclassified, unknown |
| PA0647 | | *PA0646-PA0647-PA0648* | -2.68 | Related to phage, transposon, or plasmid |
| PA0623 | | *PA0622-PA0623-PA0624* | -2.71 | Related to phage, transposon, or plasmid |
| PA4763 | *recN* | | -2.71 | DNA repair protein RecN |
| PA0644 | | *PA0643-PA0644-PA0645* | -2.72 | Related to phage, transposon, or plasmid |
| PA0910 | | | -2.72 | Hypothetical, unclassified, unknown |
| PA0616 | | *PA0614-PA0615-PA0616* | -2.73 | Related to phage, transposon, or plasmid |

| | | | | |
|---|---|---|---|---|
| PA0619 | | *PA0617-PA0618-PA0619-PA0620-PA0621* | -2.74 | Related to phage, transposon, or plasmid |
| PA2485 | | *PA2485-PA2486* | -2.77 | Hypothetical, unclassified, unknown |
| PA0626 | | *PA0625-PA0626-PA0627* | -2.78 | Related to phage, transposon, or plasmid |
| PA0620 | | *PA0617-PA0618-PA0619-PA0620-PA0621* | -2.79 | Related to phage, transposon, or plasmid |
| PA0625 | | *PA0625-PA0626-PA0627* | -2.84 | Related to phage, transposon, or plasmid |
| PA0320 | | *PA0319-PA0320* | -2.85 | Hypothetical, unclassified, unknown |
| PA0613 | | *ptrB-PA0613* | -2.87 | Hypothetical, unclassified, unknown |
| PA0629 | | *PA0628-PA0629-PA0630-PA0631-PA0632* | -2.87 | Related to phage, transposon, or plasmid |
| PA0639 | | *PA0636-PA0637-PA0638-PA0639-PA0640* | -2.93 | Related to phage, transposon, or plasmid |
| PA2486 | | *PA2485-PA2486* | -2.94 | Hypothetical, unclassified, unknown |
| PA3446 | | | -2.95 | Hypothetical, unclassified, unknown |
| PA0624 | | *PA0622-PA0623-PA0624* | -2.96 | Related to phage, transposon, or plasmid |
| PA0637 | | *PA0636-PA0637-PA0638-PA0639-PA0640* | -2.96 | Related to phage, transposon, or plasmid |
| PA3938 | | *PA3938-PA3937-PA3936* | -2.97 | Transport of small molecules |
| PA2494 | *mexF* | *mexE-mexF-oprN* | -3.01 | Resistance-Nodulation-Cell Division (RND) multidrug efflux transporter MexF |
| PA0632 | | *PA0628-PA0629-PA0630-PA0631-PA0632* | -3.06 | Related to phage, transposon, or plasmid |
| PA0635 | | *PA0633-PA0634-PA0635* | -3.13 | Related to phage, transposon, or plasmid |
| PA0645 | | *PA0643-PA0644-PA0645* | -3.13 | Related to phage, transposon, or plasmid |
| PA0627 | | *PA0625-PA0626-PA0627* | -3.15 | Related to phage, transposon, or plasmid |

| | | | | |
|---|---|---|---|---|
| PA0638 | | *PA0636-PA0637-PA0638-PA0639-PA0640* | -3.16 | Related to phage, transposon, or plasmid |
| PA0640 | | *PA0636-PA0637-PA0638-PA0639-PA0640* | -3.17 | Related to phage, transposon, or plasmid |
| PA0628 | | *PA0628-PA0629-PA0630-PA0631-PA0632* | -3.21 | Related to phage, transposon, or plasmid |
| PA0634 | | *PA0633-PA0634-PA0635* | -3.21 | Related to phage, transposon, or plasmid |
| PA0643 | | *PA0643-PA0644-PA0645* | -3.21 | Related to phage, transposon, or plasmid |
| PA0617 | | *PA0617-PA0618-PA0619-PA0620-PA0621* | -3.33 | Related to phage, transposon, or plasmid |
| PA0636 | | *PA0636-PA0637-PA0638-PA0639-PA0640* | -3.41 | Related to phage, transposon, or plasmid |
| PA0618 | | *PA0617-PA0618-PA0619-PA0620-PA0621* | -3.46 | Related to phage, transposon, or plasmid |
| PA3229 | | | -3.51 | Hypothetical, unclassified, unknown |
| PA2484 | | | -3.55 | Hypothetical, unclassified, unknown |
| PA4881 | | | -3.77 | Hypothetical, unclassified, unknown |
| PA0985 | *pyoS5* | | -3.9 | Membrane proteins/Secreted Factors (toxins, enzymes, alginate) |
| PA2491 | *mexS* | | -4.69 | Negative regulation of secondary metabolite biosynthetic process |
